# Application of T‐cell receptor repertoire as a novel monitor in dynamic tracking and assessment: A cohort‐study based on RA patients

**DOI:** 10.1111/jcmm.17623

**Published:** 2022-11-28

**Authors:** Peiqing Yang, Yijing He, Pingying Qing, Wangdong Xu, Dan Xie, Jean‐Baptiste Cazier, Xiao Liu, Csilla Varnai, Yi Zhou, Yi Zhao, Huairong Tang, Xiaoming Yin, Yi Liu

**Affiliations:** ^1^ Department of Rheumatology, West China Hospital Sichuan University Chengdu China; ^2^ Precision Medicine Key Laboratory of Sichuan Province & Precision Medicine Center West China Hospital, Sichuan University Chengdu China; ^3^ Laboratory of Nervous System Disease and Brain Functions, Clinical Research Institute The Affiliated Hospital of Southwest Medical University Luzhou China; ^4^ Department of Evidence‐Based Medicine School of Public Health, Southwest Medical University Luzhou China; ^5^ Center for Computational Biology University of Birmingham Birmingham UK; ^6^ BGI‐Shenzhen and Shenzhen Key Laboratory of Transomics Biotechnologies BGI‐Shenzhen Shenzhen China; ^7^ Department of Medical Affairs, West China Hospital Sichuan University Chengdu China; ^8^ Health Management Center West China Hospital of Sichuan University Chengdu China; ^9^ BGI Genomics BGI‐Shenzhen Shenzhen China

**Keywords:** biomarkers, DMARDs, next‐generation sequencing, rheumatoid arthritis, T‐cell receptor repertoire

## Abstract

T‐cell receptor repertoire (TCRR) sequencing has been widely applied in many fields as a novel tool. This study explored characteristics of TCRR in detail with a cohort of 598 rheumatoid arthritis (RA) patients before and after anti‐rheumatic treatments. We highlighted the abnormal TCRR distribution in RA characterized by decreased diversity and increased proportion of hyperexpanded clones (HECs), which was potentially attributed to skewed usage of global V/J segments but not a few certain ones. Enriched motifs analysis in RA community demonstrated the huge heterogeneity of CDR3 sequences, so that individual factors are strongly recommended to be taken into consideration when it comes to clinical application of TCRR. Disease‐modifying antirheumatic drugs (DMARDs) can regulate immune system through recovery of TCRR richness to relieve symptoms. Remarkably, sensitive gene profile and advantageous gene profile were identified in this study as new biomarkers for different DMARDs regimens.

## 1 INTRODUCTION

Rheumatoid arthritis (RA) is a systemic autoimmune disease characterized by chronic joint destruction and auto‐antibody production. Aberrant activation of CD4^+^ T cells plays a vital role in pathology of RA,^1^, ^2^ hence identification and quantification of CD4^+^ T cells are important to understand their role in mechanisms and develop strategies of early diagnosis and clinical intervention. T‐cell receptor repertoire (TCRR) is an innovative approach to study the biological and pathological function of T cells, and it has been applied in many fields including tumoral immunology and stem cell transplantation^3^, ^4^; however, researches of TCRR in rheumatic and autoimmune diseases are still very limited. TCRR of CD4^+^ T cells in RA patients was found less diverse than that in healthy people,^5^, ^6^, ^7^ and this aberrance was detected in both peripheral blood and inflamed synovial tissue.^8^, ^9^ Besides, abnormal distribution of TCRR was considered to play a role in the breakdown of peripheral immune tolerance.^10^ An explicit portrait of TCRR distribution in RA community is not reached yet due to the incompatible findings of previous studies: overexpression of certain V genes such as TRBV3, TRBV14, TRBV16 and TRBV17^11^, ^12^, ^13^, ^14^ was detected in different studies; however, no consensus was reached among them. Moreover, although researchers have validated the hyper‐expansion of CD4^+^ T‐cell clonotypes in both inflamed focus and peripheral blood,^14^, ^15^, ^16^ proportions of CDR3 sequences with different clonal sizes still remain undefined. Besides, enriched motifs in RA patients have not been fully explored yet.

Clinical response to disease modifying antirheumatic drugs (DMARDs) various among patients and accumulating research were carried out to dig out new biomarkers for precise medicine. Nevertheless, only a few proteins were considered to potentially predict response with unsatisfying sensitivities so far and they were not applied to clinical practice yet.^17^ Furthermore, the mechanisms of disease controlling by DMARDs treatments remain unknown. Given the important role of CD4^+^ T cells in both pathology and treatment of RA, a comprehensive understanding of CD4^+^ T‐cell biology during the DMARDs treatment course is of great necessity.

In the present study, a cohort of 598 RA patients were included to probe into characteristics of TCRR in patients with different clinical features, and detailed discussion of TCRR distribution during anti‐rheumatic therapy was applied to help understand the underlying mechanisms of DMARDs treatment and find out new biomarkers with certain specificities.

## 2 MATERIALS AND METHODS

### 2.1 Patients, cohorts and samples

Patients that met the 2010 ACR/EULAR RA diagnostic criteria were recruited from outpatient clinics of West China Hospital. All patients have given written informed consents and ethical approval was gained from Ethics Committee of Sichuan University. Individuals that had a history of any current infection, severe physical disorders including heart failure, chronic renal diseases or any type of cancer were excluded in this study. Clinical information regarding age, sex, ESR, CRP, self‐assessment score, health assessment questionnaire (HAQ), tender joint count, swollen joint count, DAS28 score and medication was recorded. For each sample, 4 ml peripheral blood was collected and restored at −80° for long‐term storage. Totally, 994 peripheral blood samples were collected for TCR sequencing and these samples consisted of the Original Cohort (OC). Samples were divided into 3 clusters based on the length of therapeutic course: Baseline was referred to samples of RA patients who had not received any anti‐rheumatic treatments before, or patients that had received any other clinical treatments recently, such as NSAIDs, antibiotics and traditional Chinese medicines, but experienced a wash‐out period for at least 3 months. Time1 included samples of RA patients who met the criterion of Baseline and had a 3‐month standard anti‐rheumatic treatment prescribed by rheumatologists from West China Hospital. Time2 contained samples of patients who met the criterion of Baseline and had a 6‐month standard anti‐rheumatic treatment. Of note, there were 23 patients in Time1 or Time2 who did not have blood collection at Baseline but met the standard of each checkpoint, therefore these patients should also be included. 176 patients in total had clinical records and blood collection at all three checkpoints, and these patients consisted of the Complete cohort (CC). Numbers of blood samples at each checkpoint were shown in Figure S1. Healthy people without any histories of RA or other chronic diseases were recruited as control cohort.^5^ All blood samples of RA patients and healthy people were processed by BGI company following the published methodology.^5^

### 2.2 Library preparation and sequencing

The gDNA of peripheral blood mononuclear cells (PBMCs) was extracted with a QIAamp DNA Blood Mini Kit. The approach to minimize the PCR bias and the mixture primers for amplifying CDR3 of TCRβ chain of CD4^+^ T cells have been described in detail in a previous research.^18^ 5 μg gDNA was collected and used as a template for multiplex PCR with an equimolar pool of 30 TCR Vβ forward primers (the VβF pool) and an equimolar pool of the 13 TCR Jβ reverse primers (the JβR pool). The reaction conditions were as follows: 1 × QIAGEN Multiplex PCR Master Mix, 0.5 × Q solution, 0.2 μM VβF pool and 0.2 μM JβR pool in a 50 μl final reaction volume. The cycling processes were as follows: 95°C for 15 min, 25–30 cycles at 94°C for 30 s, 60°C for 90 s and 72°C for 30 s, plus a final extension at 72°C for 5 min. The PCR production was loaded on a 2% agarose gel, and the 110–180 bp fractions were excised and purified.^19^ The MPCR products of prepared samples were then sequenced on BGISEQ500 platform with 200 single end reads.

### 2.3 Data processing

TCR sequencing data of CD4^+^ T cells from both RA and healthy samples were processed by IMonitor and detailed description could be found in previous work.^18^ The process is as follows: in the first step, the reads were checked for inclusion of adaptor sequences. If any adaptor sequence was detected and located within 50 bp of the 3'end of the read, it was deleted from the read. Reads bearing adaptor sequence at the 5′ end or >5% ‘N’ bases were discarded. The average base quality of each read was calculated after removing the low‐quality bases (base quality <10) at the 3′ end. Further filtration left out reads with average quality <15. Thus, low‐quality reads were removed, and adapter sequences were trimmed, and PE reads were merged to one sequence by an in‐house program and COPE^20^ subsequently. Second, the merged sequences were aligned to the reference germline sequences from IMGT^21^ database by BLAST^22^, ^23^ and further re‐aligned. Third, PCR and sequencing errors were corrected, and the CDR3 region was identified to be translated into amino acids then. Additionally, any low‐frequency sequence (CDR3 abundance<=2) was discarded to reduce the effect of sequencing error.

### 2.4 Statistical analysis

Statistical analysis was performed in R (version 3.4.4). Diversity of TCRR was assessed by Shannon entropy, Simpson Index^24^ and Diversity 50 (D50).^25^ The value of entropy was calculated by summing the frequency of each CDR3 clone with the log2 of that frequency.^26^ Shannon entropy was applied to both CDR3 sequence diversity and V/J segment diversity. Proportion of top clones was computed to assess clonality of TCRR. Pearson correlation coefficient was used to measure the linear correlation between two sets of data. Motif analysis was conducted with MEME‐chip tool^27^ (https://meme‐suite.org/meme/tools/meme‐chip), and it reports the estimated statistical significance of a motif as an E value. The E value is an estimate of the expected number of motifs with the given log likelihood ratio (or higher) and with the same width and site count that one would find in a similarly sized set of random sequences.^28^ The range of Bhattacharyya coefficient is between 0 and 1, with 0 indicating no overlap between two repertoires and 1 indicating identical TCR repertoire between two samples. The difference of clonal distribution of CDR3 sequences was displayed by the number^29^ and frequency^30^ of CDR3 sequences on different clonal size. Gene expression was calculated with the log2 fold change and shown in volcano plots.

The Wilcoxon‐Mann–Whitney test was used in comparison between two groups, and Benjamini–Hochberg correction was then applied to get the *p*‐values. *p*‐values less than 0.05 were considered statistically significant.

## 3 RESULTS

Overall, 994 samples from 598 RA patients and 439 healthy samples were included to analyse the characteristics of TCRR. Clinical information of both OC and CC is shown in Tables 1 and 2. Statistical analysis of clinical measurements of RA (ESR, CRP, HAQ, Self‐assessment score, tender joint count, swollen joint count and DAS28) have been performed. All indexes showed significant decrease in OC after treatment, and self‐assessment score, swollen joint count and DAS28 reduced significantly in CC after therapy. Sequencing information is provided in Table S1. Information of healthy cohort could be found in previous research.^5^

**TABLE 1:** Clinical Information of RA cohort

Clinical information	Baseline (*n* = 597) (Mean ± SD)	Time1 (*n* = 198) (Mean ± SD)	Time2 (*n* = 199) (Mean ± SD)	*p* value (Time1 versus Baseline)	*p* value (Time2 versus Baseline)
Age	50.28 ± 12.11	50.03 ± 11.63	49.96 ± 11.68	–	–
Female (%)	82.8%	84.32%	85.10%	–	–
ESR	38.13 ± 28.42	31.73 ± 25.48	33.80 ± 35.16	0.0275	0.0163
CRP	13.48 ± 22.73	11.92 ± 21.8	11.28 ± 22.11	0.0289	0.012
Self‐assessment score	40.01 ± 26.94	32.99 ± 24.72	31.51 ± 24.71	0.0014	0.0001
HAQ	7.43 ± 9.36	5.94 ± 8.83	6.23 ± 9.11	0.0471	ns
Tender joint count	5.59 ± 7.3	4.87 ± 6.78	4.14 ± 5.91	ns	0.0238
Swollen joint count	5.59 ± 7.3	1.26 ± 2.73	1.24 ± 4.86	0.0008	<0.0001
DAS28 score	4.11 ± 1.63	3.81 ± 1.48	3.54 ± 1.41	ns	0.0002

Abbreviations: CRP, C reacting protein; DAS28, Disease activity score 28; ESR, erythrocyte sedimentation rate; HAQ, Health assessment questionnaire; ns, not significant.

**TABLE 2:** Clinical Information of complete cohort (*n* = 176)

Clinical information	Baseline (Mean ± SD)	Time1 (Mean ± SD)	Time2 (Mean ± SD)	*p* value (Time1 vs. Baseline)	*p* value (Time2 vs. Baseline)
Age	50.11 ± 11.68			–	–
Female (%)	86.2%			–	–
ESR	34.79 ± 29.56	30.7 ± 25.94	33.79 ± 36.42	ns	ns
CRP	16.47 ± 45.48	10.37 ± 20.27	9.98 ± 19.93	ns	ns
Self‐assessment score	38.09 ± 27.27	33.25 ± 25.1	31.96 ± 25.33	0.028	0.0071
HAQ	6.48 ± 8.61	5.90 ± 8.85	6.43 ± 9.27	ns	ns
Tender joint count	4.54 ± 6.64	4.94 ± 6.84	4.06 ± 5.72	ns	ns
Swollen joint count	2.47 ± 6.08	1.12 ± 2.41	1.23 ± 5.14	0.0166	0.0001
DAS28 score	3.91 ± 1.58	3.83 ± 1.45	3.55 ± 1.40	ns	0.0181

Abbreviations: CRP, C reacting protein; DAS28, Disease activity score 28; ESR, erythrocyte sedimentation rate; HAQ, Health assessment questionnaire; ns, not significant.

### 3.1 TCRR diversity of RA patients is significantly decreased compared with healthy cohort

**FIGURE 1:**
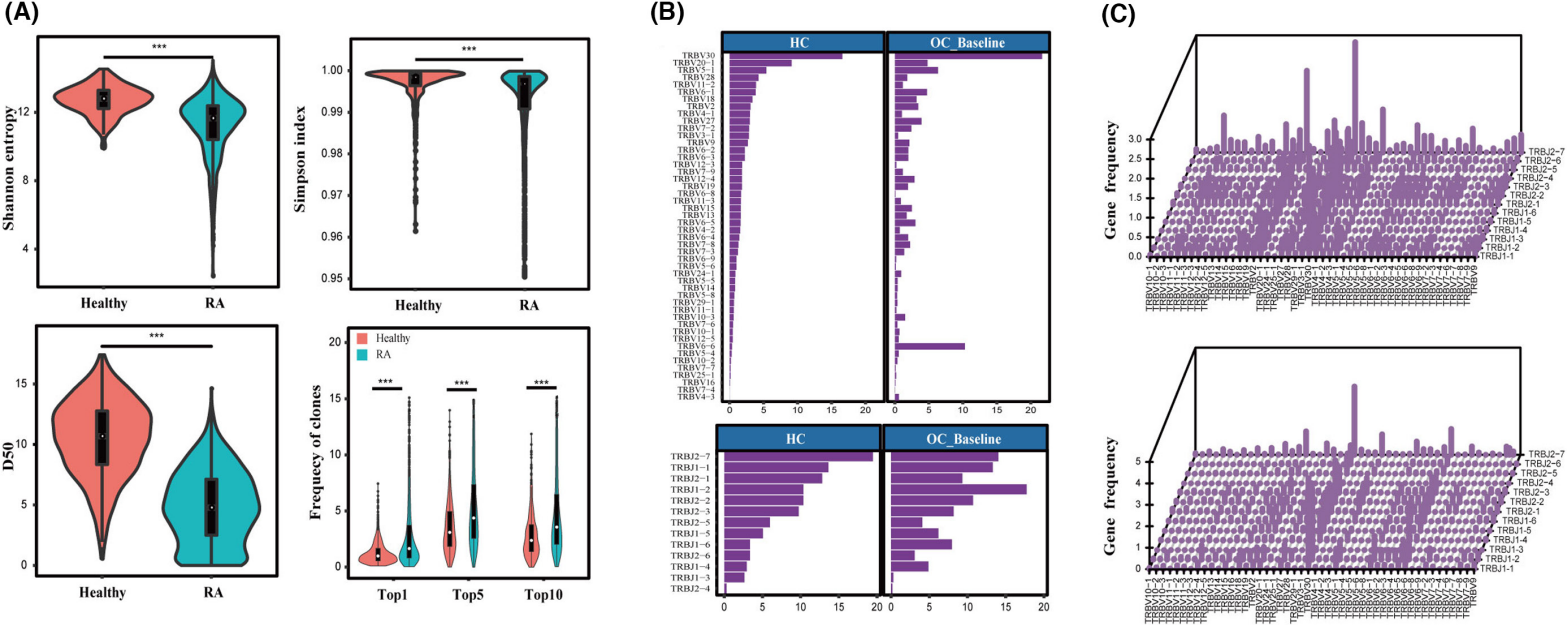
TCRR distribution in HC and OC Baseline RA patients. (A) TCRR diversity analysis in HC and RA. (B) V/J gene usage frequency in HC and RA. (C) V‐J paie usage frequency in HC (upper) and RA (lower). Gene usage frequency (GUF) was calculated based on clonal reads. Significant difference of GUF: V genes: 45 (45 in total); J genes: 12 (13 in total, except for TRBJ2‐2); V‐J pairs: 577 (625 in total). Statistic values could be found in Tables S2–S4. TCRR: T‐cell receptor repertoire; HC, Healthy cohort; OC, Original cohort

Several studies reported CD4^+^ T‐cell repertoire of RA patients with a limited number of patients^31^, ^32^; however, a full‐scale elaboration of TCRR features with large masses of samples and data are still urgently needed given the variety of T cells in physiological circumstance. Hence, we specified the repertoire presentation of CD4^+^ T cells in 597 patients from OC Baseline to address the changes and abnormality of TCRR in RA (Figure 1). CDR3 diversity in RA Baseline was significantly lower than that in HC as reflected by Shannon entropy (*p* = 6.53 e‐69), D50 (*p* = 6.72 e‐127) and Simpson index (*p* = 2.33 e‐36). By contrast, percentages of Top1/5/10 clones were significantly higher in Baseline (*p*(Top1) = −2.905801 e‐33; *p*(Top5) = −1.844825 e‐30; *p*(Top10) = −6.400133 e‐29), indicating that TCRR of RA patients possessed higher clonality.

The amino acid structure of CDR3 sequences is mainly dependent on the rearrangement of V and J gene segments, determining the specific response to HLA complex and antigenic peptides.^33^ To spotlight the distribution of V/J genes of CD4^+^ T cells in RA, we checked the gene usage frequency (GUF, calculated by clonal reads) of TRBV/TRBJ segments and V‐J pairs (Figure 1B,C) and compared them statistically with healthy cohort (Tables S2‐S4). GUF of all 45 V genes, 12 out of 13 J genes (except for TRBJ2‐2) and 577 out of 625 V‐J gene pairs showed significant difference in Baseline from HC, respectively, which demonstrated that abnormal gene expression took place in global V/J genes but not certain ones. Furthermore, the peak of CDR3 sequence length in RA was slightly lower than that in HC (Figure S1), implying that length distribution of CDR3 in RA patients was abnormally shorter than the healthy one. The different length distribution was reported to play a role in biased TCRR with accumulation of auto‐reactive T cells^34^; so, we concluded that skewed CDR3 length was probably related to the development of RA. The discoveries underscored that TCRR diversity of RA patients presented significantly lower diversity and higher clonality compared with healthy people, which is partly attributed to a wide range of V/J segments and V‐J pairs expressing abnormal frequency.

### 3.2 TCRR diversity is negatively correlated with disease activity

**FIGURE 2:**
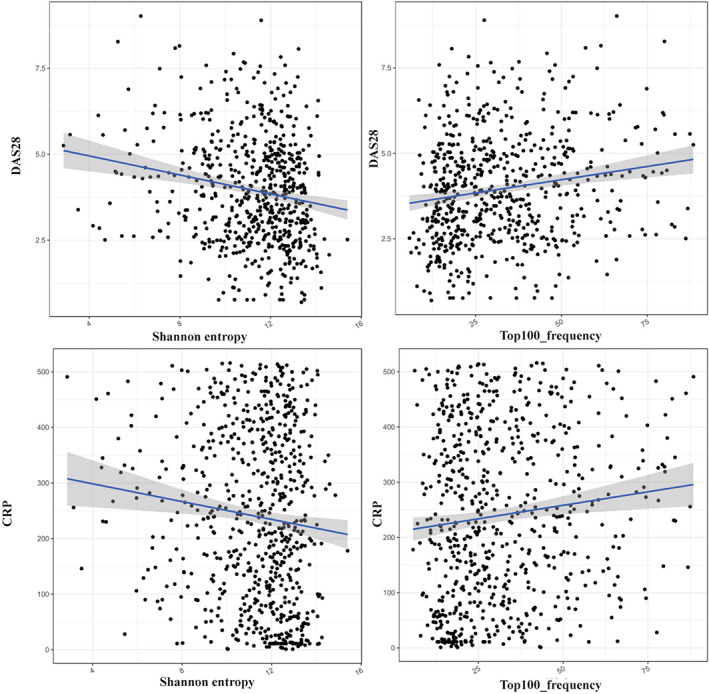
Correlation analysis of DAS28‐Shannon entropy, DAS‐Top100 frequency, CRP‐Shannon entropy and CRP‐Top100 frequency. CRP, C‐reacting protein; DAS: Disease activity score

DAS28 is a continuous measure of RA disease activity that combines information from swollen joints, tender joints, acute phase response and general health. DAS28‐CRP and DAS28‐ESR are widely used for clinical evaluation in RA management. Some research reported a correlation between disease activity and TCRR diversity in RA,^5^, ^6^ and we further examined the relevance with detailed discussion to highlight the influence of inflammatory reaction on the repertoires. First, correlation analysis was conducted in 4 groups followed as DAS28 and shannon entropy, DAS28 and Top100 frequency, C reacting protein (CRP) and shannon entropy, as well as CRP and Top100 frequency (Figure 2). Poisson coefficients were displayed as −0.179 (DAS28‐Shannon entropy, *p* = 0.01), 0.170 (DASA28‐Top100 frequency, *p* = 0.005), −0.107 (CRP‐shannon entropy, *p* = 0.005) and 0.110 (CRP‐Top100 frequency, *p* = 0.004). The results illustrated that there was negative correlation between DAS28/CRP and TCRR diversity, and a positive correlation was identified between DAS28/CRP and Top100 ratio. In brief, TCRR showed declining diversity and increased clonality as the level of systemic inflammation rise.

**FIGURE 3:**
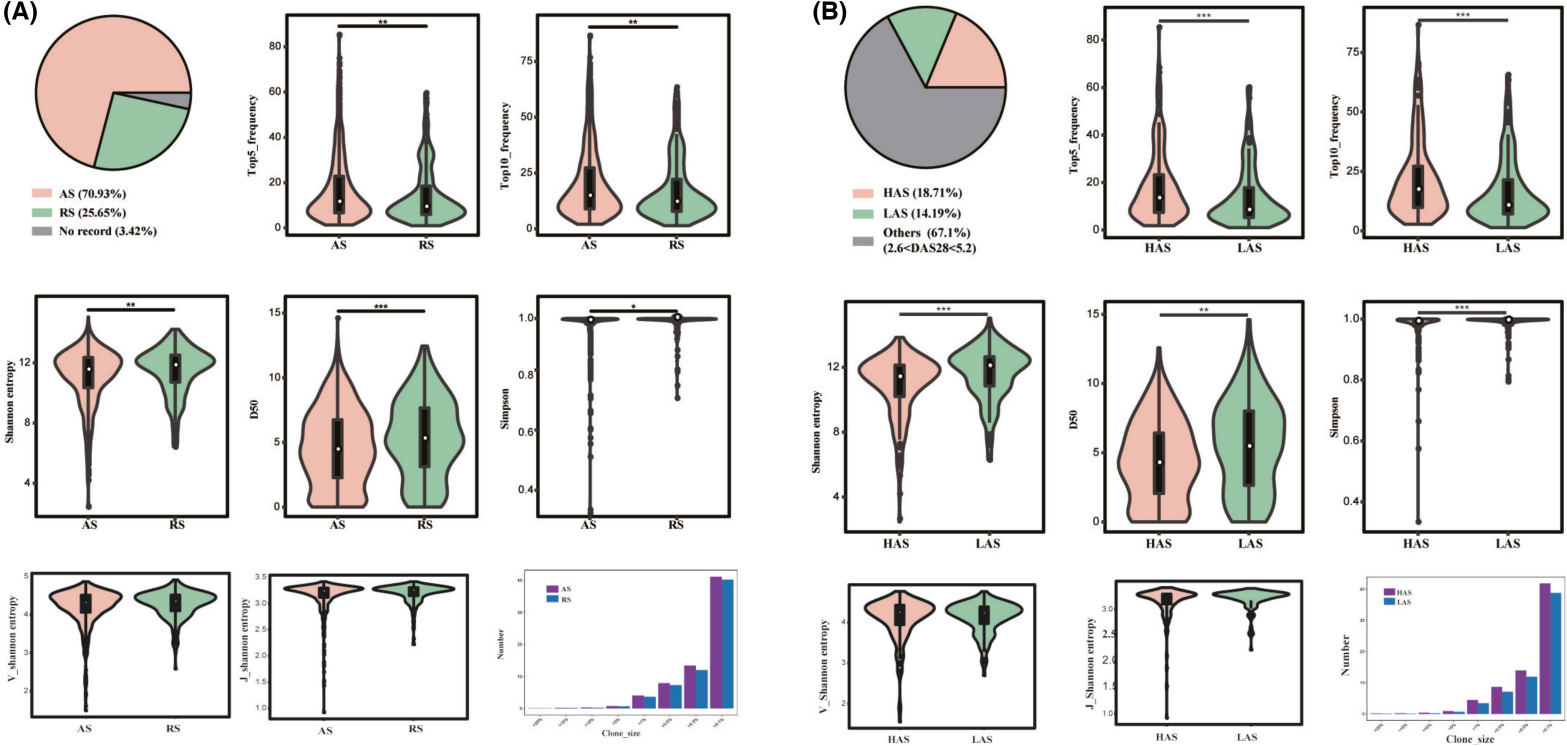
TCRR diversity analysis in patients with different levels of disease activity. (A) Comparison of TCRR diversity between AS and RS; (B) Comparison of TCRR diversity between HAS and LAS. AS, Active stage; HAS, High activity stage; LAS, Low activity stage; RS, Remission stage

To further underline how considerable is the difference of TCRR diversity in patients with various clinical manifestations, comparisons were performed between groups of RA patients with different DAS28 scores. Among patients in OC Baseline, there were 705 (70.93%) in active stage (DAS28 score >2.6, AS) and 255 (25.65%) in remission stage (DAS28 score <2.6, RS). For patients in AS, there were 186 (18.71%) in high activity stage (DAS28 score >5.1, HAS) and 141 (14.19%) in low activity stage (2.6 < DAS28 score <3.2, LAS). Overall, CDR3 diversity of AS was significantly higher than that of RS (Figure 3A): the value of Shannon entropy (*p* = 0.007), Simpson (*p* = 0.0129) and D50 (*p* = 0.0005) decreased significantly whereas the proportion of Top5 (*p* = 0.0068) and Top10 (*p* = 0.0026) clones increased significantly in AS. The size of Top50 clones in both AS and RS was shown in Figure 3A. The number of sequences with a clonal size between 0.1% and 0.3% of the total volume is the largest, and number of CDR3 clones with any clonal size in AS was higher than that in RS. Furthermore, no significant difference of V/J gene diversity (based on Shanoon Entropy) was observed (*p*(V) = 0.17, *p*(J) = 0.13). The results indicated that TCRR in HAS group had stronger clonality but less diversity. Similarly, CDR3 diversity of HAS group significantly differed from that of LAS (Figure 3B, Shannon entropy (*p* = 0.00041); D50 (*p* = 0.00629); Simpson (*p* = 0.0006); Top5 (*p* = 0.0006); Top10 (*p* = 0.0002)) and the number of Top50 clones with any clonal size in HAS was larger than that in LAS. Moreover, no significant difference was detected in V/J Shannon entropy (*p*(V) = 0.72, *p*(J) = 0.29). These results elucidated that TCRR of LAS was more diverse. Our results highlighted the noticeable difference of repertoires distribution between patients with mild symptoms and patients with severe symptoms using a wider range of diversity indicators. Sum all the findings up, we deduced that TCRR diversity was considerably different in patients with different levels of inflammation, and patients with high DAS28 scores had a higher proportion of top clones, which may imply the existence of prominent clonal proliferation of disease associated CD4^+^ T cells.

### 3.3 No difference of TCRR diversity was found between ANA (+) and ANA (−) RA patients

Antinuclear antibody (ANA) is found to be crucial to many autoimmune diseases such as systemic lupus erythematosus, however, the association between ANA and development of RA is not thoroughly underlined. Hence, we compared the diversity of repertoires between ANA positive samples and ANA negative samples. Among 182 patients (27.16%) from Baseline who have a clear record of ANA test in the past 6 months, 90 (13.48%) were ANA positive and 92 (13.68%) were ANA negative. No significant difference was observed in any comparisons (Figure S2), which did not suggest a strong link between the existence of ANA and higher/lower TCRR diversity. These results may also provide an explanation of the positive rate of ANA in RA patients, which is only around 30%–50%.^35^ Intriguingly, another study reported a higher repertoire diversity measured by the unique clone number in ANA positive RA patients,^5^ which is inconsistent with our finding that no significant difference of unique CDR3 number was found between the two groups. More research is still needed to give further clarification of the connection between them.

### 3.4 Clonal size distribution and motif analysis

**FIGURE 4:**
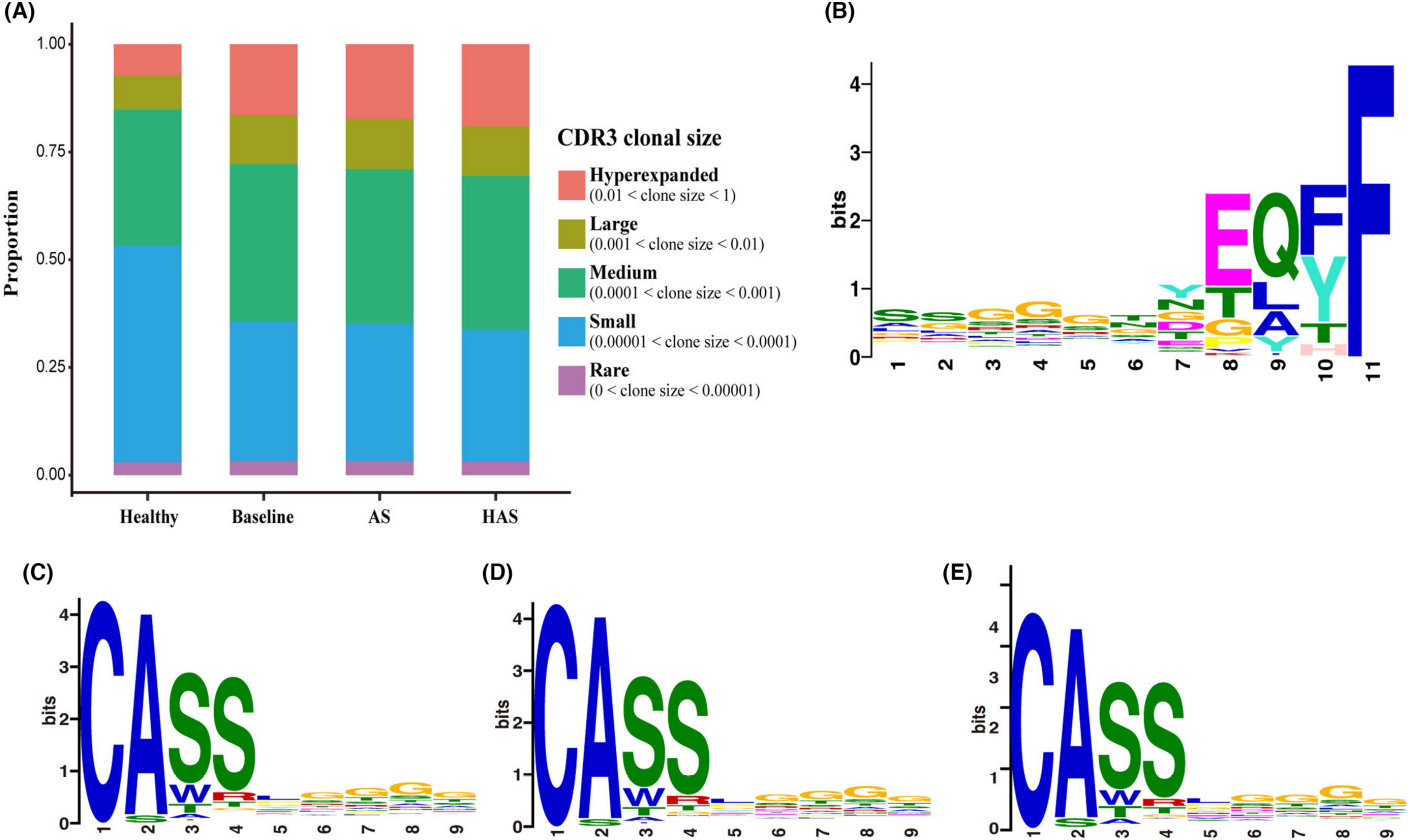
Analysis of size and sharing of CDR3 clones. (A) CDR3 clonal size distribution in HC, Baseline, AS and HAS; (B–E) Enriched motif analysis by MEME‐chip in HC (B), Baseline (C), AS (D) and HAS (E). AS, Active stage; HAS, High activity stage; HC, Healthy control

To analyse the basis of diversity loss, all CDR3 sequences from OC Baseline patients and HC were grouped into 5 clusters based on clonal abundance: hyperexpanded, large, medium, small and rare (Figure 4A). Columns showed the occupancy of clonotypes in frequency of the overall TCRR. It is clear that percentages of hyperexpanded clones (HECs) and large clones were much higher in RA patients than HC, while a reduction in small clones was visible. It also showed that as disease activity score rise, proportion of hyperexpanded clones HECs increased, which might be the reason for increased clonality, and severer clinical symptoms as discussed above. HECs were reported to accumulate in both synovium and peripheral blood of RA patients and were considered to dominate antigen‐specific T‐cell response.^36^, ^37^, ^38^, ^39^ To have a better understanding of these sequences, we extracted all 1373 HECs from 547 samples and compared them with 52 published RA‐associated CDR3 clones.^5^ We found 6 CDR3 clones that were overlapped in both cohorts as follows: CASSLDGKGYTF/ CASSLTDVAYEQYF/ CASSSRLAGGTDTQYF/ CASSYSGNDEQYF/ CATSRGTVSYEQYF/ CATSRVAGETQYF.

We conducted enriched motif analysis by MEME‐chip in HC, OC Baseline, AS and HAS, to fill the gap of motif investigation in CDR3 of pathological CD4^+^ T cells in RA. MEME‐chip analysis distinguished RA successfully from HC, highlighting a clustering pattern CASS enriched in the N‐terminus of β chain in Baseline, AS and HAS (E (Baseline) = 4.4 e‐7777, E (AS) = 9.6 e‐5753, E (HAS) = 2.8 e‐1700, respectively. Figure 4B–E). For healthy people, most enriched motif was found in the C‐terminus showing as EQFF/EQYF (E (HC) = 1.1 e‐2912). CASS cluster was not considered to dominate antigen‐specific T‐cell responses of RA since it tends to be conserved and was detected in other circumstances such as virus infection^40^; however, the results strongly implied that CDR3 sequences for RA‐specific antigens were highly diversified among individual repertoires given that antigen recognition does not require the complete CDR3.^41^ The noticeable heterogeneity of CDR3 clones gives a span‐new perspective to understand the composition of CD4^+^ T cells repertoires and the condition of immune system in RA patients. Furthermore, it also underlines the feasibility of clinical application of TCR sequencing with the notion of individual TCRR profile in terms of disease monitoring and assessment.

### 3.5 DMARDs treatment increased richness of TCRR

**FIGURE 5:**
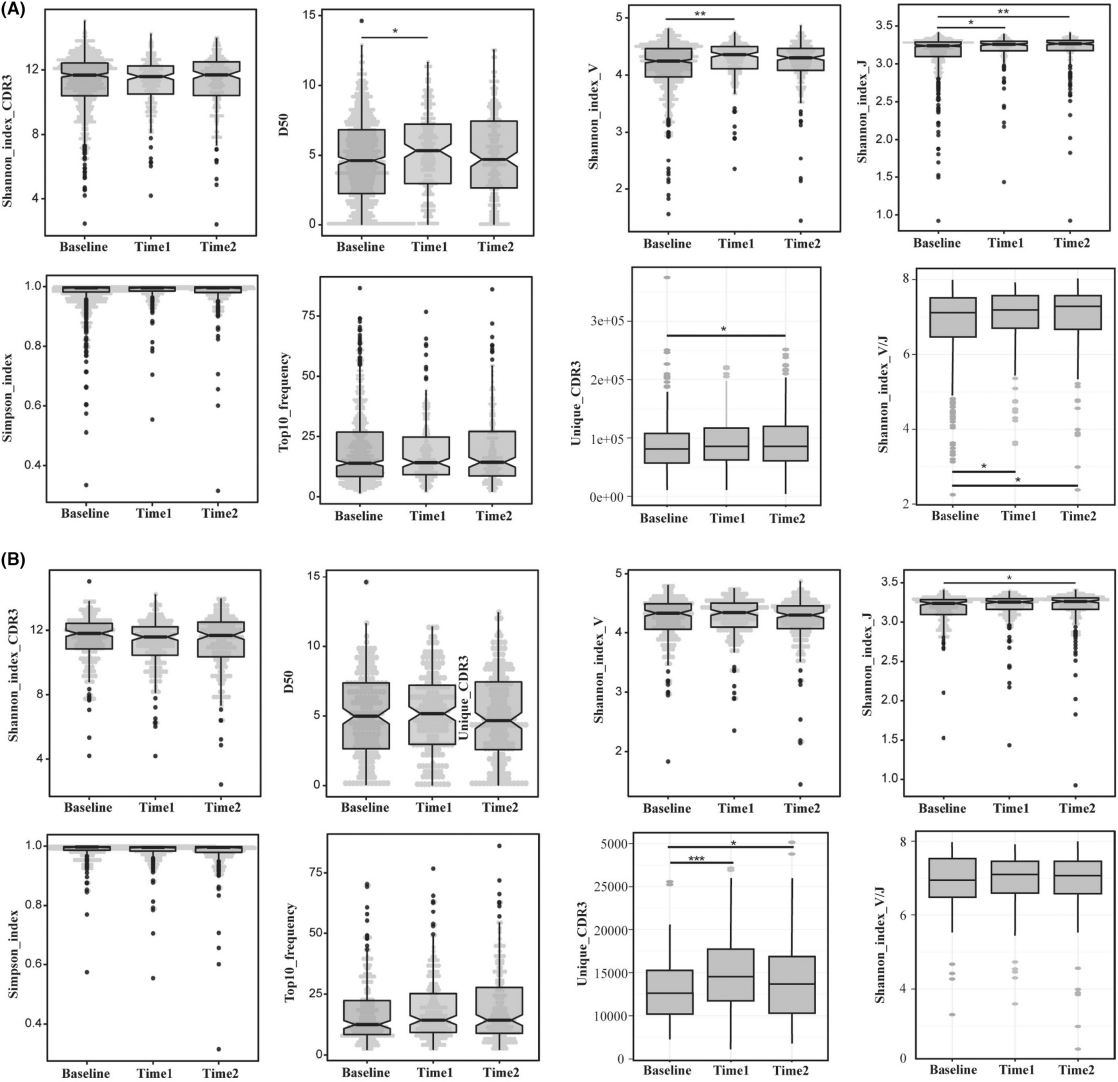
Dynamic TCRR tracking in OC and CC during anti‐rheumatic treatment. (A) Analysis of TCRR diversity/richness and V/J gene diversity in OC; (B) Analysis of TCRR diversity/richness and V/J gene diversity in CC. TCRR: T‐cell receptor repertoire; OC: Original Cohort; CC: Complete Cohort. (Wilcoxon signed‐rank test, *p*‐value < 0.05*, *p*‐value < 0.005**, *p*‐value < 0.001***)

DMARDs refer to drugs that interfere with signs and symptoms of RA, improves physical function and inhibits progression of joint damage, nevertheless, the principle of DMARDs therapy still remains unclear. In this section, we discussed the potential mechanisms of DMARDs treatment through alterations of TCRR during therapeutic course. Overall, DAS28 decreased significantly in both OC (*p* = 0.0002, Time2 versus Baseline) and CC (*p* = 0.0181, Time2 versus Baseline) after treatment, which suggests considerable efficacy of DMARDs therapy. Dynamic changes of TCRR diversity in two cohorts were shown in Figure 5. In OC, significant increase of D50 (*p* = 0.0249) at Time1 was observed and unique CDR3 had a significant growth at Time2 (*p* = 0.027). Other indicators of CDR3 diversity did not show significant alterations. To be clear, anti‐rheumatic therapy revived CDR3 richness and enlarged the categories of different CDR3 sequences in OC patients. There were 43 genes in total that displayed significant changes after treatment (Figure 3). Both V(*p* = 0.001) and J(*p* = 0.02) genes' Shannon entropy significantly elevated at Time1, and J genes' diversity increased at Time2 (*p* = 0.001) and V‐J pairing diversity augmented at both Time1 (*p* = 0.044) and Time2 (*p* = 0.018).

Similar results were found in the analysis of CC (Figure 5B): unique CDR3 ascended significantly at both Time1 (*p* = 0.0003) and Time2 (*p* = 0.015), and CDR3 diversity remained unchanged after treatment as reflected in all indicators. There was a significant increase of J gene's diversity at Time2 (*p* = 0.02); while no changes of V genes' frequency were detected either in either Time1 or Time2. The remarkable increase of gene diversity is in consistence with the recovery of CDR3 richness since re‐arrangement of V(D)J segments determines the CDR3 sequence structure. Based on all above, we believed that DMARDs treatment is capable of reviving CDR3 richness through the recovery of V/J gene diversity to strength the immune system and deactivate inflammation. Besides, we inferred that compared with V genes, recovery of J genes would be more stable and detectable in the application of monitoring and assessing curative effect.

### 3.6 New biomarkers for DMARDs treatment

To further highlight the changes of TCRR distribution in RA patients treated with different DMARDs, 3 subgroups were set up from CC based on different regimens: 80 patients (50.64%) treated with MTX (methotrexate); 71 patients (45.51%) treated with MTX and glucocorticoid (GC) and 5 patients (3.85%) treated with etanercept (TNF inhibitor, TNFi). First, we analysed dynamic alterations of repertoire diversity in each group during treatment. CDR3 diversity did not show significant changes over time in any group, and it was coincident with outcomes of CDR3 diversity analysis in total CC samples. To find out whether there is a difference of recovering CDR3 diversity between any two regimens, comparisons were made between MTX and MTX + GC, MTX and TNFi, as well as MTX + GC and TNFi at Baseline, Time1 and Time2, respectively. However, no significant difference of CDR3 diversity was observed in any comparisons above (Figure S4).

**FIGURE 6:**
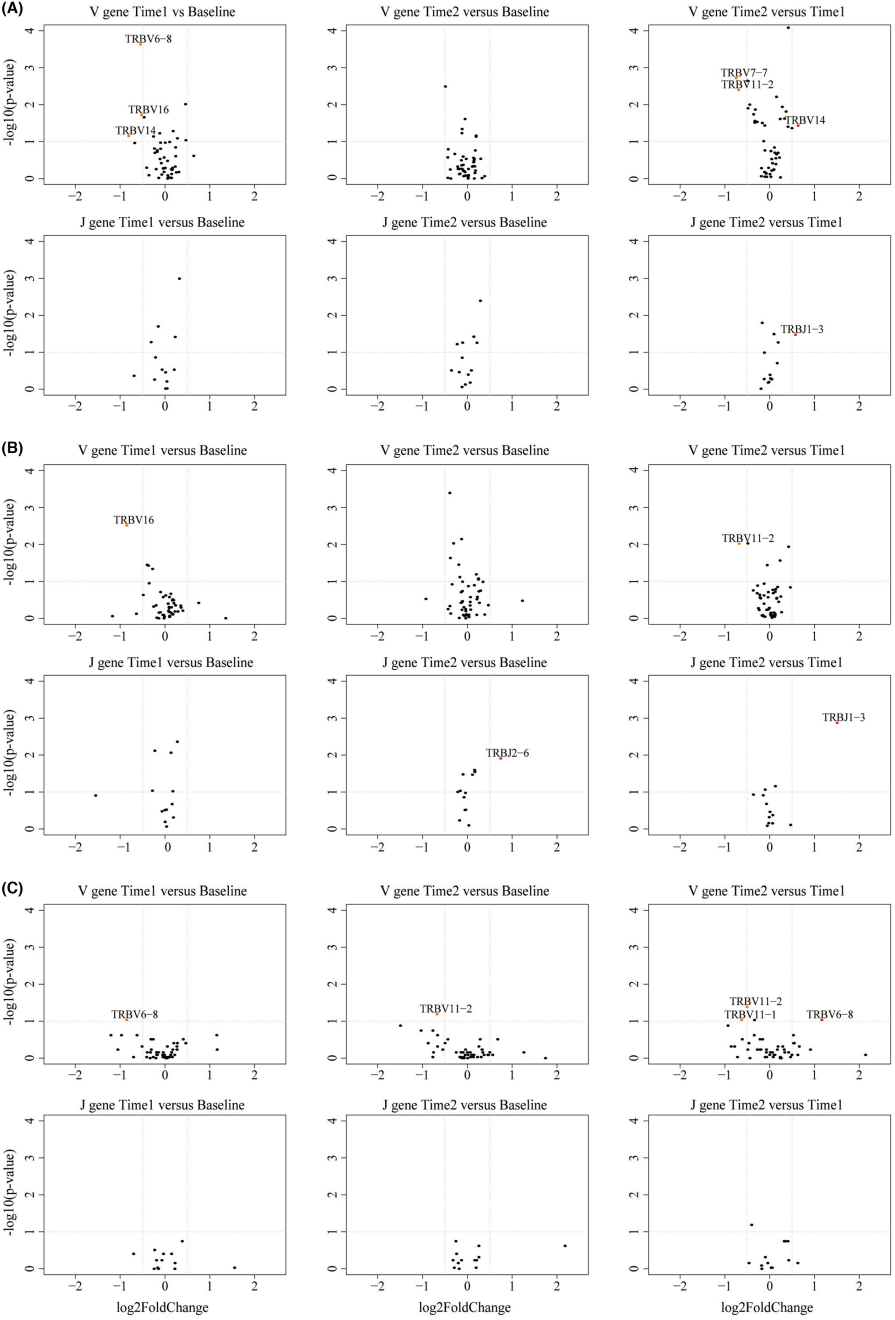
Comparison of gene usage frequency at different times for RA patients treated with MTX (A), MTX + GC (B) and TNFi (C). Horizontal axis was marked with log2 fold change and vertical axis was marked with ‐log10 of *p* value.

By contrast, significant changes of V/J GUF were detected (Figure 6). For dynamic tracking of gene expression in MTX group, 3 genes (TRBV6‐8/TRBV16/TRBV14) showed lower GUF at Time1 compared with the Baseline, and 2 genes (TRBV7‐7/TRBV11‐2) presented significantly decreased GUF at Time2 compared with Time1. GUF of TRBJ1‐3 and TRBV14 increased significantly at Time2 compared with Time1 (Figure 6A). Based on the values of GUF in Baseline and HC, it was concluded that MTX recovered TRBV14, TRBV16 and TRBJ1‐3 effectively. In MTX + GC group, GUF of TRBV16 declined significantly at Time1 compared with Baseline and TRBV11‐2 displayed a reduced GUF at Time2 compared with Time1; while GUF of TRBJ2‐6 rose significantly at Time2 compared with Baseline and TRBJ1‐3's GUF increased at Time2 compared with Time1 (Figure 6B). Totally, MTX + GC was able to restore the GUF of TRBV16, TRBJ2‐6 and TRBJ1‐3. In TNFi group, significant decrease was observed in the GUF of TRBV6‐6 (Time1 vs. Baseline), TRBV11‐2 (Time2 vs. Baseline), TRBV11‐1 and TRBV11‐2 (Time2 vs. Time1). GUF of TRBV6‐8 raised up significantly at Time2 compared with Time1 (Figure 6C). It was clear that TRBV6‐8 was retrieved by TNFi specifically. These recovered genes are considered to be sensitive indicators of curative efficacy, on the basis of which, we summarized sensitive gene profiles for MTX, MTX + GC and TNFi, respectively (Table 3).

**TABLE 3:** Gene profile of different DMARDs

DMARDs	Sensitive genes	Advantageous genes
MTX	TRBV14，TRBV16， TRBJ1‐3	TRBV12‐5，TRBV28， TRBV20‐1，TRBJ2‐6
MTX + GC	TRBV16，TRBJ2‐6， TRBJ1‐3	TRBV28，TRBV20‐1， TRBJ2‐6
TNFi	TRBV6‐8	TRBV3‐1，TRBV4‐1， TRBV10‐2

Abbreviations: DMARDs, Disease‐modifying antirheumatic drug; GC, glucocorticoid; MTX, methotrexate; TNFi, tumour necrosis factor inhibitor.

**FIGURE 7:**
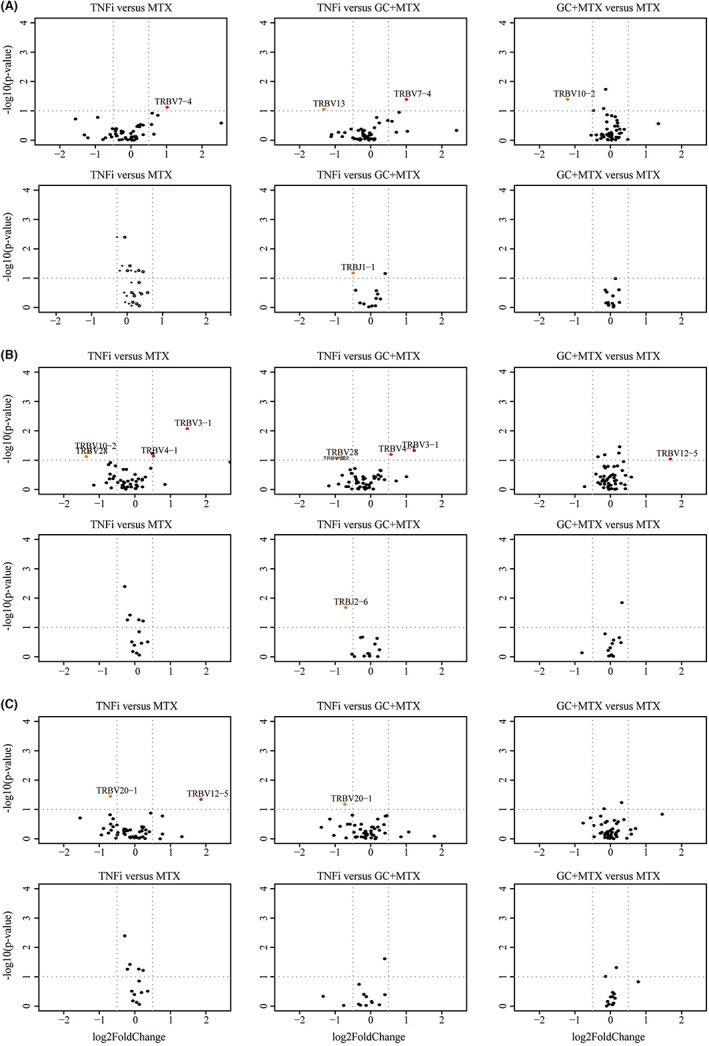
Comparison of gene usage frequency between different therapy groups in Baseline (A), Time1 (B) and Time2 (C). Horizontal axis was marked with log2 fold change and vertical axis was marked with ‐log10 of *p* value.

The ability of every regimen to revive V/J genes was also compared at the same checkpoint in MTX versus MTX + GC, MTX versus TNFi and MTX + GC versus TNFi (Figure 7). First, we evaluated the variation in each comparison at Baseline: no prominent heterogeneity was detected and only 4 genes' GUF (TRBV7‐4, TRBV13, TRBV10‐2 and TRBJ1‐1) was different before treatment (Figure 7A); so, we presumed that the rest of V/J genes were quite comparable. At Time1, GUF of TRBV3‐1 and TRBV4‐1 was significantly higher in biologics group, and TRBV10‐2 and TRBV28 showed significantly lower GUF compared with both MTX and MTX + GC group. Besides, expression of TRBJ2‐6 in MTX + GC group was higher than biologics group. TRBV12‐5's displayed increased GUF in MTX + GC group compared with MTX group. At Time2, TRBV12‐5's GUF was significantly higher in biologics group than MTX group, while GUF of TRBV20‐1 in biologics group dropped significantly compared with other 2 groups. These findings implied that there was significant difference in the abilities of each DMARDs regimen to regulate V/J genes' expression and each regimen literally had its own preponderant genes which reacted better to corresponding treatments. Based on comparisons above, advantageous genes of MTX, MTX + GC and TNFi were summarized in Table 3, respectively.

## 4 DISCUSSION

In this study, we reported the characteristics of T‐cell receptor repertoire in a cohort of 598 RA patients. The etiological factors of RA are various and complicated, among which autoreactive CD4^+^ T‐cell response is considered to play a key role. To the best of our knowledge, this study included the largest number of patients in TCR repertoire sequencing research of rheumatic diseases, which promises the credibility of results and convincing evidence. The discoveries of this study that differ from most previous researches are reflected in the following points: first, a detailed portrait of TCRR distribution of peripheral CD4 + T cells in RA patients was reported with a wide range of V/J genes showing biased GUF; second, enriched motif analysis based on disease activity showing the notable heterogeneity of CDR3 sequences in RA community; third, explanation of therapeutic mechanisms of DMARDs from a particular perspective; moreover, the clinical application of TCRR functioning as a dynamic monitor is recommended; last but not least, we highlighted potentially new biomarkers for DMARDs regimens.

Research have reported that the diversity of TCRR is reduced in some autoimmune diseases due to the specific autoantigen‐derived amplification of T‐cell clones, and our results highlighted that decreased diversity of TCRR is partly attributed to global alteration in TCRR V/J gene pool. To be more specific, more than 90% of these genes or gene pairs showed skewed expression, which is in contradictory of previous view that TCRβV gene usage was variably skewed to certain genes.^13^, ^14^, ^42^, ^43^, ^44^ The extensive aberration observed in V/J segments' expression is supported by another study which also included a large amount of RA patients,^5^ emphasizing the significance of sufficient sample size to improve reliability of TCRR analysis. Increased clonality was also observed in repertoires of CD4^+^ T cells in RA patients. The over‐proliferation of Top1/5/10 clones is potentially owned to the continuous stimulation of antigen exposure^38^ and specifically, aberrant gene rearrangements disrupt the normal establishment of immune repertoire with the existence of auto‐antigen irritation, leading to over‐proliferation of disease‐specific clones. These clonal types themselves become new antigens which give birth to a vicious circle of the expansion of unwonted clonotypes.^15^ Take the extensive bias in V/J gene pool into consideration, there should be numerous types of CDR3 sequences, and our results verified this heterogeneity given that no enriched non‐conserved motifs were found. We consider it as a circumstantial proof for the existence of multi‐autoantigens in pathological environment of RA, which might explain the existence of various profiles of TCRR presentation. In this connection, individual differences should be greatly taken into account for future work instead of focusing on similarity or commonality of CDR3 clones. Moreover, setting up personal TCRR profile is highly recommended for both clinical assessment and dynamic tracking of disease development.

Enriched motifs of RA patients in different diseases activity groups basically remained the same, visualized as the conserved N‐terminal amino acid residues CASS; however, they were significantly different from that of healthy people (conserved C‐terminal residues EQFF/EQYF). On the one hand, it could be deduced that both structure and position of enriched terminal amino acid residues are influenced by specific autoantigens in RA patients. On the contrary, amino acid sequence of CDR3 in non‐conserved region is highly variable even in RA patients sharing most similar clinical symptoms, implying the great heterogeneity of CDR3 clones. It is worth noting that 6 HECs showed over‐expression in RA population compared with healthy people. Even though each clone was detected only once in community, this finding still brought out two points to note that first, the structure of amino acid arrangement in HECs may vary widely in different cohorts; moreover, the 6 shared HECs were expected to potentially provide ideas for RA‐specific biomarkers exploration since they were detectable in two different cohorts and considered to be disease‐related.

The main goal of RA clinical management is to terminate inflammation, relieve symptoms, improve social function and reduce long‐term complications. As far as we have known, our study is the first to discuss the mechanisms of DMARDs with the application of dynamic TCR sequencing data in considerable numbers of RA patients receiving DMARDs treatments. This survey indicates that DMARDs treatment is capable of reviving TCRR richness instead of CDR3 diversity, which is concordant with the increased diversity of V/J segments after treatment. Hence, the mechanisms of DMARDs therapy could be explained as follows: immune system is modulated and boosted after treatments via the expansion of variety of CDR3 sequences to control inflammation and alleviate conditions. It is also noticed that the recovery of J genes was easier and more stable to detect than V genes; therefore, we presume that diversity of J genes is potential to function as a reliable monitor in assessing therapeutic effects.

MTX therapy was proved to result in an increase in the level of apoptosis in transformed T cells, the extent of which is dependent on the dose and duration of treatment.^45^ It could be seen that GUF of TRBV‐14 changed significantly in MTX treatment group. Biologic functions of TRBV14 are considered to relate to phenotypes of immune system, such as T‐cell differentiation and abnormality in T‐cell number (NCBI Entrez Gene and GeneCards database). We hypothesize that the phenotype and the number of pathogenic T cells are potentially regulated by MTX through TRBV14, thus controlling and reducing the level of inflammation. Besides, GUF of TRBV16, TRBJ1‐3 and TRBJ2‐6 was restored in both MTX and MTX + GC group, while recovery of TRBJ2‐6 was detected only in MTX + GC. This finding might suggest the sensitivity of TRBJ2‐6 to GC treatment. Our results also showed that GUF of TRBV6‐8 fluctuated in TNFi (etanercept) treatment group, which suggested that TRBV6‐8 reacted better to TNFi than other gene segments. Noticeably, we highlighted sensitive gene profile and advantageous gene profile of MTX, MTX + GC and etanercept, respectively. Sensitive gene profile reflects intrinsic early changes in TCRR gene pool of each DMARDs regimen, therefore is of great value in helping clinicians estimate the therapeutic efficiency. Advantageous gene profile, on the contrary, represents the preponderant target genes of csDMARDs and bDMARDs. To be more specific, TRBV3‐1/TRBV4‐1/TRBV10‐2 may have a better response to etanercept, while TRBV28/TRBV12‐5/TRBV20‐1/TRBJ2‐6 respond better to traditional DMARDs such as MTX and GC. The finding that TRBV28 insufficiently responds to TNFi is supported by another research, which proved that recovery of TRBV28 by single TNFi was not satisfying.^6^ Advantageous gene profile functions as a ‘orienting biomarker’ for different DMARDs, so that a proper treatment strategy could be determined as early as possible based on personal TCRR profile. One limitation of this study is that only 5 patients were included in TNFi group, so further research including more samples will be of great value. Besides, since this is a single‐country, single‐centre study, racial differences might have effects on results. In summary, this study provides a new perspective for understanding the pathology of RA and boosts the application of TCR sequencing in both diagnosis and treatment for RA patients.

## AUTHOR CONTRIBUTIONS

**Peiqing Yang:** Conceptualization (lead); data curation (supporting); formal analysis (supporting); investigation (equal); project administration (equal); resources (supporting); writing – original draft (equal). **Yijing He:** Data curation (equal); formal analysis (lead); methodology (supporting); project administration (supporting); software (equal); validation (supporting); visualization (lead). **Pingying Qing:** Funding acquisition (supporting); investigation (supporting); project administration (supporting); resources (supporting). **Wangdong Xu:** Data curation (equal); project administration (supporting); resources (lead). **Dan Xie:** Data curation (supporting); formal analysis (equal); methodology (equal); supervision (equal). **Jean‐Baptiste Cazier:** Formal analysis (supporting); methodology (supporting); supervision (equal); validation (lead); visualization (supporting); writing – review and editing (equal). **Xiao Liu:** Methodology (lead); supervision (supporting). **Csilla Varnai:** Formal analysis (supporting); methodology (supporting); software (supporting). **Yi Zhou:** Project administration (supporting); resources (equal). **Yi Zhao:** Conceptualization (supporting); project administration (supporting). **Huairong Tang:** Project administration (supporting); resources (supporting). **Xiaoming Yin:** Project administration (supporting); resources (supporting). **Yi Liu:** Conceptualization (lead); funding acquisition (lead); project administration (supporting); resources (lead); writing – review and editing (equal).

## CONFLICT OF INTEREST

The authors declared that they have no conflicts of interest to this work.

## Supporting information


FigureS1

FigureS2

FigureS3

FigureS4

TableS1

TableS2

TableS3

TableS4

## Data Availability

Research data are not available due to data missing.
