# Supplementary material for: Application of T‐cell receptor repertoire as a novel monitor in dynamic tracking and assessment: A cohort‐study based on RA patients
Source: J Cell Mol Med. 2022 Nov 28;26(24):6042–55. doi: 10.1111/jcmm.17623 (PMC9753462; doi:10.1111/jcmm.17623)
Supplement: Supplementary file 1 — FigureS1 [file JCMM-26-6042-s002.pdf]

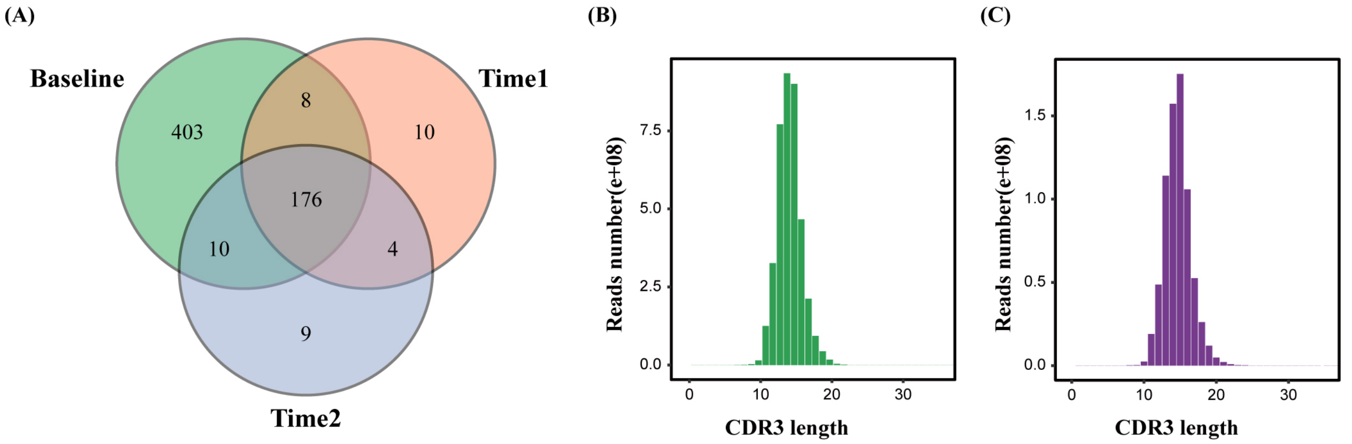

Supplement Figure 1 Number of RA samples collected at OC Baseline, Time1 and Time2 (A) and CDR3 length distribution in RA samples (B) and HC (C)
