# Supplementary figures and images for: Application of T‐cell receptor repertoire as a novel monitor in dynamic tracking and assessment: A cohort‐study based on RA patients
Source: J Cell Mol Med. 2022 Nov 28;26(24):6042–55. doi: 10.1111/jcmm.17623 (PMC9753462; doi:10.1111/jcmm.17623)

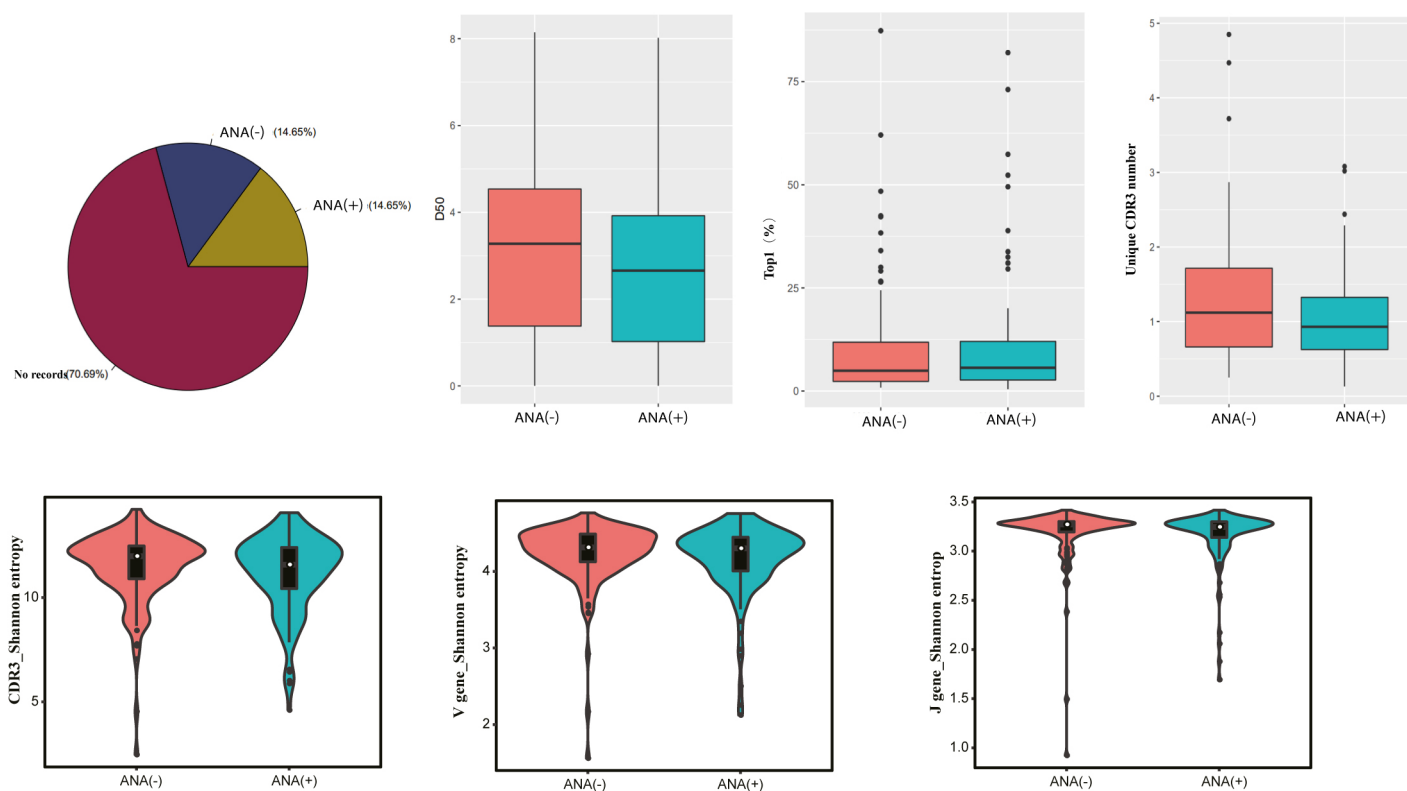

**Supplement Figure 2** TCRR diversity analysis in ANA(-) patients and ANA(+) patients.

Supplement: Supplementary file 2 — FigureS2 [file JCMM-26-6042-s006.pdf]

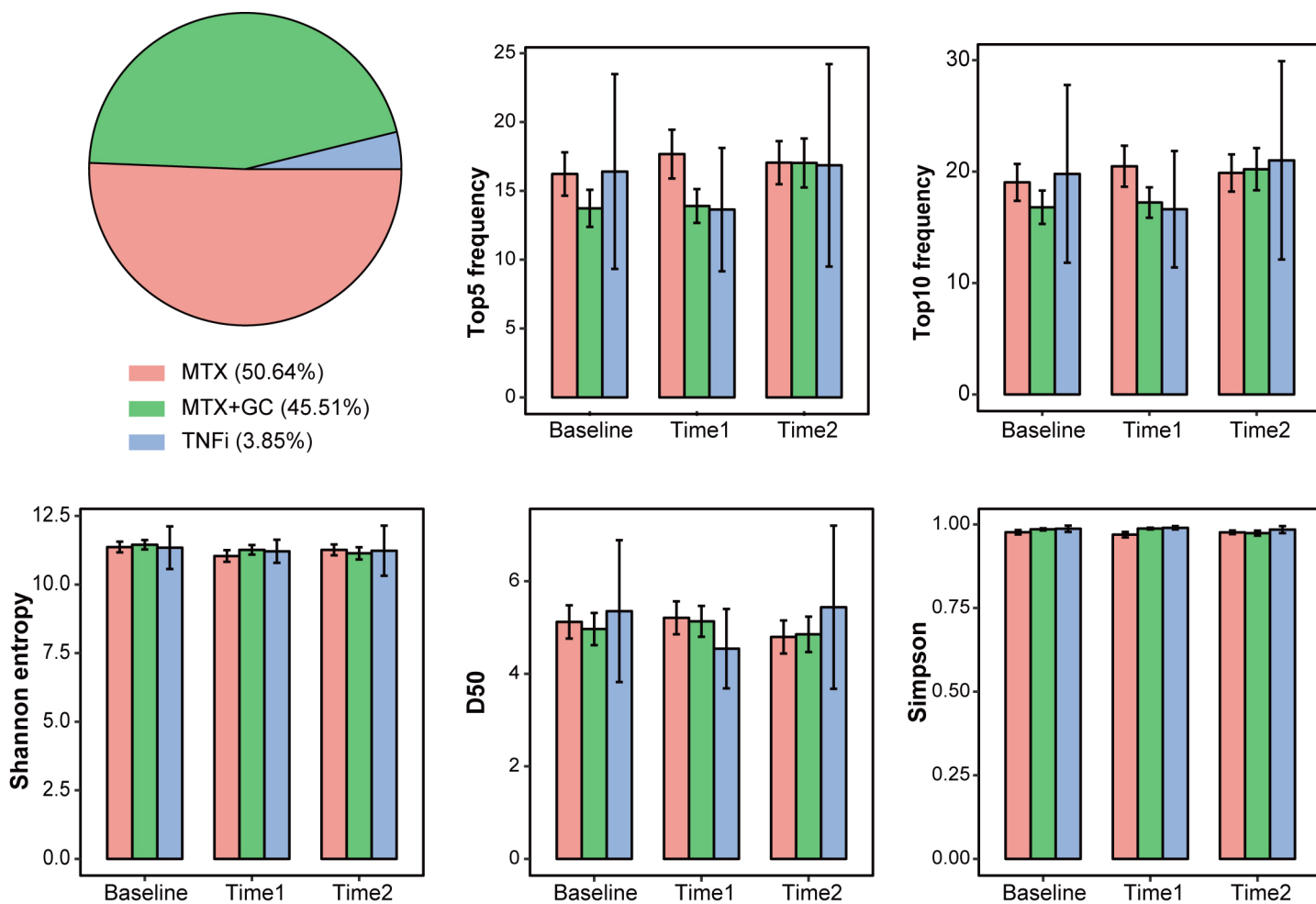

**Supplement Figure 4 CDR3 diversity of MTX, MTX+GC and TNFi at different checkpoints**

Supplement: Supplementary file 4 — FigureS4 [file JCMM-26-6042-s007.pdf]
