# Supplementary material for: Application of T‐cell receptor repertoire as a novel monitor in dynamic tracking and assessment: A cohort‐study based on RA patients
Source: J Cell Mol Med. 2022 Nov 28;26(24):6042–55. doi: 10.1111/jcmm.17623 (PMC9753462; doi:10.1111/jcmm.17623)
Supplement: Supplementary file 3 — FigureS3 [file JCMM-26-6042-s004.pdf]

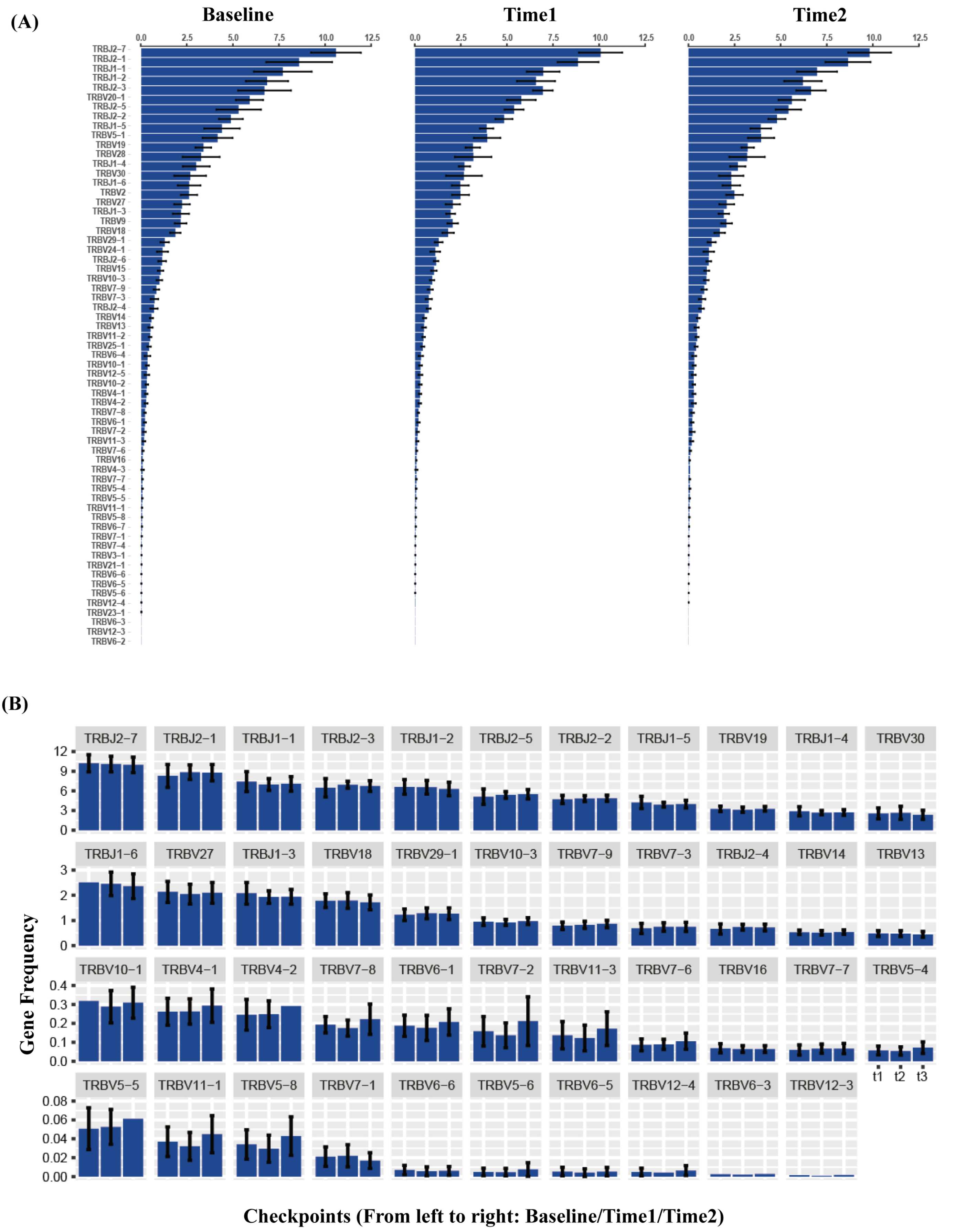

Supplement Figure 3 Dynamic expression of V/J genes with significant changes after DMARDs therapy
