# Supplementary material for: Application of T‐cell receptor repertoire as a novel monitor in dynamic tracking and assessment: A cohort‐study based on RA patients
Source: J Cell Mol Med. 2022 Nov 28;26(24):6042–55. doi: 10.1111/jcmm.17623 (PMC9753462; doi:10.1111/jcmm.17623)
Supplement: Supplementary file 5 — TableS1 [file JCMM-26-6042-s008.pdf]

Supplement Table 1 Sequencing quality control of RA blood samples

Of Features	Mean	Minimum	Maximum	Median
**Top1 clone frequency (100%)**	9.02	0.29	82.73	4.61
**Top5 clones frequency (100%)**	16.37	0.99	85.35	10.94
**Top10 clones frequency (100%)**	19.39	1.41	86.58	14.07
**NO. of CDR3 reads (10^6)**	3.94	0.57	14.42	3.67
**Unique CDR3 reads (10^4)**	1.74	0.29	6.21	1.61
