# Supplementary material for: Application of T‐cell receptor repertoire as a novel monitor in dynamic tracking and assessment: A cohort‐study based on RA patients
Source: J Cell Mol Med. 2022 Nov 28;26(24):6042–55. doi: 10.1111/jcmm.17623 (PMC9753462; doi:10.1111/jcmm.17623)
Supplement: Supplementary file 6 — TableS2 [file JCMM-26-6042-s003.pdf]

Supplement Table 2

	Baseline_mean	Baseline_median	Baseline_upperQuantile	Baseline_lowQuantile	HC_mean	HC_median	HC_upperQuantile	HC_lowQuantile	P value	W-value	LFC
TRBV10-1	0.67022321	0.47830731	0.69844017	0.33018375	0.46015047	0.41252	0.52333189	0.30483036	1.77E-08	177508	0.54253594
TRBV10-2	0.37487877	0.23965483	0.35289677	0.16333108	0.17922962	0.154347	0.20274473	0.11421652	1.97E-41	120818	1.06461507
TRBV10-3	1.50974352	1.27592638	1.7288904	0.98411716	0.59391434	0.5316448	0.65446556	0.45104927	1.82E-144	33369	1.3459767
TRBV11-1	0.22507013	0.17369686	0.23585942	0.12747725	0.61412213	0.53373573	0.64126739	0.45038607	1.40E-174	421598	-1.448151
TRBV11-2	1.15305034	0.88856343	1.23878996	0.62825232	3.87658812	3.68238064	4.22482219	3.20523759	8.95E-182	425796	-1.749332
TRBV11-3	0.87053152	0.68193147	1.00841942	0.4559186	1.66771145	1.21387808	2.21379681	0.88630794	1.72E-73	349132	-0.9379012
TRBV12-3	0.22808051	0.2007584	0.27909704	0.14040789	1.82689235	1.64455912	2.06087762	1.32915351	3.03E-199	435654	-3.0017766
TRBV12-4	2.87824039	2.44057355	3.4432242	1.65028742	1.81238762	1.65930466	2.03849256	1.36365553	4.87E-39	123800	0.66729555
TRBV12-5	0.56941801	0.30481509	0.51953448	0.18241641	0.44297511	0.36909248	0.523597	0.24796105	2.38E-05	248702	0.36226248
TRBV13	1.72457205	0.94222606	1.36141792	0.65111088	1.62778246	1.27346418	1.6758569	1.02251607	1.02E-28	298473	0.0833305
TRBV14	0.38716415	0.25680241	0.34533558	0.18578443	0.78096459	0.61377583	0.72991477	0.51251488	1.22E-142	401807	-1.0123118
TRBV15	2.50393755	1.83066396	2.60171519	1.25769161	1.63466686	1.34410968	1.68010356	1.10696399	2.79E-20	151554	0.61520194
TRBV16	0.08846291	0.03064342	0.04966371	0.01922749	0.05176712	0.02682206	0.0387822	0.01921738	0.00068506	193663	0.77303656
TRBV18	3.18441865	2.7496341	4.19767403	1.51836613	3.34905456	3.30222291	4.16297031	2.32941087	3.48E-07	254977	-0.0727239
TRBV19	1.93132176	1.64536421	2.29490692	1.17607227	1.80335366	1.68521019	2.00864599	1.4136589	0.33823108	225099	0.09890619
TRBV2	3.43082594	2.96882596	3.90009232	2.19970621	3.07053619	2.87084515	3.42893959	2.4237567	0.5125022	213453	0.16006533
TRBV20-1	4.79184878	4.2710668	5.62391513	3.21136295	9.12453815	8.93639642	10.1620395	7.89630029	1.70E-154	409393	-0.9291692
TRBV24-1	0.91345178	0.62541431	0.95064075	0.43877269	0.85202542	0.6902747	0.87674217	0.54385897	0.00097907	241988	0.10043211
TRBV25-1	0.18486241	0.14157523	0.20567145	0.09420492	0.14509076	0.10887368	0.16615845	0.0691858	3.08E-11	170215	0.34949624
TRBV27	3.90460748	3.13342613	4.30483738	2.25603855	2.96402483	2.67898994	3.28038027	2.09911316	4.79E-09	175907	0.39762
TRBV28	1.80742964	1.18107705	1.92698828	0.78967461	4.24859182	4.08722248	5.33281185	2.94190519	7.73E-123	388391	-1.2330453
TRBV29-1	0.38047119	0.30645099	0.44746857	0.19800186	0.6289145	0.59452386	0.69050266	0.50159191	1.23E-97	369612	-0.7250767
TRBV30	21.671294	20.7372485	28.6721439	12.7814678	16.587579	16.8238028	20.3616489	12.8414381	2.70E-14	163214	0.38568196
TRBV3-1	0.47054806	0.34643419	0.52448519	0.19781441	2.83859533	2.62596065	3.24078597	2.08505191	2.30E-190	430700	-2.5927632
TRBV4-1	1.03555835	0.8486047	1.18608739	0.53479802	2.98345647	2.7388898	3.39292542	2.25200278	1.12E-165	416280	-1.5265759
TRBV4-2	0.72655308	0.58676531	0.81120839	0.37985974	1.54666352	1.38256568	1.73958495	1.14385591	3.38E-140	400206	-1.0900193
TRBV4-3	0.57115049	0.23744295	0.75979417	0.04574636	1.26082436	1.1959558	1.92588036	0.00295198	5.23E-05	239825	-1.1424245
TRBV5-1	6.33936134	5.73995796	7.88298035	3.77510305	5.40636722	5.23795283	6.06270243	4.43947544	0.00076193	193874	0.229678
TRBV5-4	0.55313212	0.41187215	0.55223315	0.27771409	0.28578223	0.25313103	0.31120845	0.20932186	2.04E-52	108160	0.9527079

TRBV5-5	0.41294933	0.34461873	0.4648984	0.2373218	0.7986336	0.71905253	0.84272011	0.61748356	8.08E-134	395956	-0.951569
TRBV5-6	0.13405389	0.08652577	0.13185626	0.05497939	0.98979073	0.82387233	1.02200262	0.68503733	7.50E-188	429277	-2.8843105
TRBV5-8	0.33382457	0.25403849	0.34049426	0.17940537	0.6851899	0.62784491	0.76171547	0.53240772	4.45E-151	407237	-1.0374137
TRBV6-1	4.71715419	3.92303059	5.42291203	2.87263839	3.82958649	3.5721991	4.48640364	2.88241493	0.00023204	191599	0.30072814
TRBV6-2	1.98010518	1.56173542	2.41034768	0.98931446	2.24373374	2.1905384	2.53714056	1.83402793	3.30E-30	300654	-0.1803244
TRBV6-3	1.97989949	1.56256115	2.41464509	0.99262124	2.2428862	2.18916318	2.53536062	1.84377234	3.13E-30	300687	-0.1799292
TRBV6-4	1.95180499	1.67585811	2.3341673	1.23270592	1.41129984	1.20620047	1.73605131	0.83035082	5.68E-26	142075	0.46778439
TRBV6-5	3.02166287	2.56336331	3.53137675	1.93875813	1.60840949	1.5402714	1.852556	1.28574635	1.27E-94	69157	0.90970796
TRBV6-6	10.2998254	8.92524627	12.2215884	7.10760366	0.35064156	0.31306152	0.40319022	0.2520608	1.55E-200	1	4.87647909
TRBV6-8	0.09119172	0.0704344	0.12570491	0.03779219	1.68222308	1.58419378	1.90581097	1.33213977	1.81E-200	436328	-4.2053224
TRBV6-9	0.20007261	0.17376795	0.23845654	0.12363859	1.00317888	0.93852987	1.17100863	0.78401604	2.70E-196	434021	-2.3259833
TRBV7-2	2.40822047	1.99825445	3.06119086	1.33172809	2.85628752	2.66583566	3.50036511	1.7285139	9.20E-13	269757	-0.2461737
TRBV7-3	1.36340598	1.13531493	1.59126933	0.79946423	1.21604446	1.11690732	1.32903218	0.89861251	0.19450719	208814	0.16501924
TRBV7-4	0.03197049	0.02453305	0.03512825	0.01621644	0.01943201	0.00885881	0.0155634	0.00468721	1.67E-80	76296	0.71830564
TRBV7-6	0.37679746	0.25591099	0.37493289	0.1682969	0.5155837	0.46935495	0.57581303	0.38014723	1.72E-83	357976	-0.4524174
TRBV7-7	0.10156258	0.0686144	0.10431281	0.04476616	0.14889599	0.11599608	0.14743431	0.08957873	1.66E-53	329382	-0.551936
TRBV7-8	2.23303963	1.93582196	2.38271939	1.45515929	1.27748636	1.19102585	1.38283273	1.0418192	1.92E-78	82782	0.80570098
TRBV7-9	1.16320162	1.03217707	1.30481801	0.81007398	1.82282467	1.72692693	2.04064197	1.5185573	3.72E-123	388615	-0.6480746
TRBV9	2.11904942	1.49380911	2.12937727	1.07974046	2.67423827	2.4214619	2.87617141	2.10960424	2.86E-70	346154	-0.3357108
