# Supplementary material for: Application of T‐cell receptor repertoire as a novel monitor in dynamic tracking and assessment: A cohort‐study based on RA patients
Source: J Cell Mol Med. 2022 Nov 28;26(24):6042–55. doi: 10.1111/jcmm.17623 (PMC9753462; doi:10.1111/jcmm.17623)
Supplement: Supplementary file 7 — TableS3 [file JCMM-26-6042-s005.pdf]

Supplement Table 3

	Baseline_mean	Baseline_median	Baseline_upperQuantile	Baseline_lowQuantile	HC_mean	HC_median	HC_upperQuantile	HC_lowQuantile	P value	W-value	LFC
TRBJ1-1	13.3182263	12.1517782	14.6917337	10.038036	13.6272367	13.4773229	14.9981728	11.7594612	1.27E-11	267082	-0.0330911
TRBJ1-2	17.7256701	16.6850423	20.162604	13.6539747	10.358888	10.0859658	11.6348392	8.74303431	6.44E-121	49333	0.77497104
TRBJ1-3	0.30006835	0.04142423	0.19990196	0.02019042	2.64710833	2.456887	2.82185243	2.16561668	1.68E-176	422727	-3.1410542
TRBJ1-4	4.88724063	4.41591844	5.57241347	3.28493741	2.93322369	2.78934617	3.23444582	2.40430417	1.67E-65	94750	0.73653305
TRBJ1-5	6.18158715	5.73859762	7.16875804	4.54498093	5.06579752	4.85559748	5.48757388	4.36809639	5.20E-20	152038	0.28718789
TRBJ1-6	7.96494676	6.36406822	8.62612387	5.09614486	3.35950639	3.25326357	3.64599922	2.91942984	8.46E-154	27411	1.24541544
TRBJ2-1	9.32483878	8.67428278	10.8723073	6.81879811	12.8175628	12.6229123	14.2667116	11.4952684	8.26E-85	359102	-0.4589713
TRBJ2-2	10.7290698	10.0533711	11.95682	8.02252063	10.3533491	9.92390461	11.1216634	9.08885127	0.47475096	223345	0.05142747
TRBJ2-3	8.18085408	7.83348716	9.28223541	6.36824472	9.73981073	9.39005651	10.2864595	8.59717186	1.04E-43	318304	-0.2516423
TRBJ2-4	0.17209244	0.13418728	0.17729049	0.09604243	0.29902133	0.25825621	0.30063431	0.21382801	1.65E-112	380956	-0.7970647
TRBJ2-5	4.09534279	3.83223856	4.79473167	3.10264305	5.9767869	5.80106964	6.48948423	5.21881526	2.99E-105	375522	-0.5453859
TRBJ2-6	3.0867255	2.62432735	3.25545331	2.04997114	3.35645437	3.12620861	3.62914468	2.75180135	2.22E-29	299450	-0.1208608
TRBJ2-7	14.0333373	13.6442457	16.1383385	11.0351162	19.4652542	19.7655329	21.4819663	17.6405306	2.82E-100	371687	-0.472043
