# Supplementary material for: Application of T‐cell receptor repertoire as a novel monitor in dynamic tracking and assessment: A cohort‐study based on RA patients
Source: J Cell Mol Med. 2022 Nov 28;26(24):6042–55. doi: 10.1111/jcmm.17623 (PMC9753462; doi:10.1111/jcmm.17623)
Supplement: Supplementary file 8 — TableS4 [file JCMM-26-6042-s001.pdf]

Supplement Table 4

V-J pair	FC	P value	FDR
TRBV10-1_TRBJ1-1	1.11730887	0.18204875	0.19124314
TRBV10-1_TRBJ1-2	2.36823488	5.17E-76	1.21E-75
TRBV10-1_TRBJ1-3	0.06504978	8.82E-151	3.88E-150
TRBV10-1_TRBJ1-4	7.07900907	1.07E-11	1.41E-11
TRBV10-1_TRBJ1-5	1.24254362	0.00206876	0.00235138
TRBV10-1_TRBJ1-6	8.73126594	5.72E-99	1.62E-98
TRBV10-1_TRBJ2-1	1.96665949	0.19166503	0.20066943
TRBV10-1_TRBJ2-2	1.20192278	0.02883632	0.03123935
TRBV10-1_TRBJ2-3	1.09752312	0.11448783	0.12149728
TRBV10-1_TRBJ2-4	0.70818294	0.00010443	0.00012202
TRBV10-1_TRBJ2-5	0.78391067	1.17E-06	1.43E-06
TRBV10-1_TRBJ2-6	1.20891513	0.45669556	0.46871386
TRBV10-1_TRBJ2-7	0.87472061	0.00022355	0.00025929
TRBV10-2_TRBJ1-1	3.72845067	7.26E-07	8.90E-07
TRBV10-2_TRBJ1-2	3.13391852	1.10E-93	2.99E-93
TRBV10-2_TRBJ1-3	0.07156842	1.89E-133	6.77E-133
TRBV10-2_TRBJ1-4	2.6645669	4.14E-20	6.13E-20
TRBV10-2_TRBJ1-5	1.91048661	2.58E-19	3.76E-19
TRBV10-2_TRBJ1-6	9.67496677	1.25E-83	3.19E-83
TRBV10-2_TRBJ2-1	1.81525625	1.03E-05	1.23E-05
TRBV10-2_TRBJ2-2	1.37711544	2.18E-15	3.04E-15
TRBV10-2_TRBJ2-3	1.59942462	4.74E-35	8.12E-35
TRBV10-2_TRBJ2-4	6.01075883	7.33E-15	1.01E-14
TRBV10-2_TRBJ2-5	0.74668143	1.82E-06	2.20E-06
TRBV10-2_TRBJ2-6	1.89821908	7.82E-12	1.03E-11
TRBV10-2_TRBJ2-7	1.04866856	0.32444606	0.33630289
TRBV10-3_TRBJ1-1	2.04534042	3.59E-83	9.07E-83
TRBV10-3_TRBJ1-2	4.01592522	1.46E-172	9.23E-172
TRBV10-3_TRBJ1-3	0.13980386	2.23E-166	1.24E-165
TRBV10-3_TRBJ1-4	4.6973102	4.46E-90	1.18E-89
TRBV10-3_TRBJ1-5	2.81882866	9.41E-112	2.84E-111
TRBV10-3_TRBJ1-6	8.63402182	9.16E-179	6.72E-178
TRBV10-3_TRBJ2-1	2.43945784	1.11E-73	2.55E-73
TRBV10-3_TRBJ2-2	3.04720399	3.02E-107	8.94E-107
TRBV10-3_TRBJ2-3	1.92528837	6.00E-65	1.31E-64
TRBV10-3_TRBJ2-4	1.87810525	3.10E-20	4.60E-20
TRBV10-3_TRBJ2-5	1.93820131	8.35E-20	1.23E-19
TRBV10-3_TRBJ2-6	1.81407181	9.33E-29	1.49E-28
TRBV10-3_TRBJ2-7	1.54378458	1.41E-30	2.32E-30
TRBV11-1_TRBJ1-1	0.2846289	5.60E-147	2.33E-146

TRBV11-1_TRBJ1-2	0.93214024	0.70451664	0.71366621
TRBV11-1_TRBJ1-3	0.05334077	2.46E-124	8.07E-124
TRBV11-1_TRBJ1-4	0.62967099	4.68E-15	6.46E-15
TRBV11-1_TRBJ1-5	0.62626727	7.55E-07	9.23E-07
TRBV11-1_TRBJ1-6	3.80898734	6.59E-43	1.21E-42
TRBV11-1_TRBJ2-1	0.22478934	1.05E-176	7.43E-176
TRBV11-1_TRBJ2-2	0.29541907	7.80E-131	2.69E-130
TRBV11-1_TRBJ2-3	0.34715024	1.01E-160	5.24E-160
TRBV11-1_TRBJ2-4	0.14804899	8.24E-05	9.66E-05
TRBV11-1_TRBJ2-5	0.13750671	4.27E-178	3.10E-177
TRBV11-1_TRBJ2-6	0.28461772	2.38E-44	4.45E-44
TRBV11-1_TRBJ2-7	0.20209161	3.49E-176	2.39E-175
TRBV11-2_TRBJ1-1	0.34316302	5.83E-170	3.43E-169
TRBV11-2_TRBJ1-2	0.40071854	7.34E-146	2.99E-145
TRBV11-2_TRBJ1-3	0.02690148	7.00E-203	4.37E-201
TRBV11-2_TRBJ1-4	0.49154767	9.50E-79	2.33E-78
TRBV11-2_TRBJ1-5	0.4382303	1.96E-125	6.51E-125
TRBV11-2_TRBJ1-6	0.39973707	2.76E-122	8.89E-122
TRBV11-2_TRBJ2-1	0.15443882	2.49E-192	3.17E-191
TRBV11-2_TRBJ2-2	0.45628467	4.79E-168	2.74E-167
TRBV11-2_TRBJ2-3	0.28724999	6.29E-184	5.23E-183
TRBV11-2_TRBJ2-4	0.10121135	1.80E-136	6.60E-136
TRBV11-2_TRBJ2-5	0.20976642	7.31E-186	6.74E-185
TRBV11-2_TRBJ2-6	0.21238851	5.06E-170	3.03E-169
TRBV11-2_TRBJ2-7	0.28194177	2.20E-175	1.49E-174
TRBV11-3_TRBJ1-1	0.63407872	4.17E-29	6.73E-29
TRBV11-3_TRBJ1-2	1.35153267	6.80E-37	1.18E-36
TRBV11-3_TRBJ1-3	0.08292613	1.93E-156	9.26E-156
TRBV11-3_TRBJ1-4	1.17223385	0.75635608	0.7649371
TRBV11-3_TRBJ1-5	2.19839491	9.98E-19	1.45E-18
TRBV11-3_TRBJ1-6	0.85640145	0.01696404	0.01857116
TRBV11-3_TRBJ2-1	0.29770213	8.32E-158	4.12E-157
TRBV11-3_TRBJ2-2	0.50532124	2.29E-92	6.18E-92
TRBV11-3_TRBJ2-3	0.37732505	4.46E-116	1.36E-115
TRBV11-3_TRBJ2-4	0.30903885	2.42E-10	3.11E-10
TRBV11-3_TRBJ2-5	0.33918839	1.95E-117	6.05E-117
TRBV11-3_TRBJ2-6	0.46476963	9.04E-35	1.55E-34
TRBV11-3_TRBJ2-7	0.45622687	6.78E-55	1.38E-54
TRBV12-3_TRBJ1-1	0.06548331	6.04E-198	1.40E-196
TRBV12-3_TRBJ1-2	0.24423623	3.22E-180	2.51E-179
TRBV12-3_TRBJ1-3	0.00705856	5.23E-229	1.09E-226
TRBV12-3_TRBJ1-4	0.1401626	7.54E-186	6.82E-185

TRBV12-3_TRBJ1-5	0.08992387	1.09E-197	2.34E-196
TRBV12-3_TRBJ1-6	2.12325366	5.23E-39	9.28E-39
TRBV12-3_TRBJ2-1	0.05109678	1.45E-198	3.78E-197
TRBV12-3_TRBJ2-2	0.08818948	6.55E-196	1.02E-194
TRBV12-3_TRBJ2-3	0.07608642	2.73E-193	3.78E-192
TRBV12-3_TRBJ2-4	0.12275255	4.64E-153	2.14E-152
TRBV12-3_TRBJ2-5	0.0646912	5.05E-197	9.01E-196
TRBV12-3_TRBJ2-6	0.06345833	1.66E-196	2.81E-195
TRBV12-3_TRBJ2-7	0.05067857	1.81E-198	4.51E-197
TRBV12-4_TRBJ1-1	1.25252111	0.00033361	0.00038479
TRBV12-4_TRBJ1-2	3.0652537	5.50E-140	2.13E-139
TRBV12-4_TRBJ1-3	0.13878402	2.04E-167	1.15E-166
TRBV12-4_TRBJ1-4	2.19436665	5.53E-101	1.58E-100
TRBV12-4_TRBJ1-5	1.29574016	1.97E-06	2.37E-06
TRBV12-4_TRBJ1-6	9.92014146	6.27E-186	6.02E-185
TRBV12-4_TRBJ2-1	0.79769472	1.43E-25	2.22E-25
TRBV12-4_TRBJ2-2	2.06403187	3.04E-53	6.17E-53
TRBV12-4_TRBJ2-3	1.21209525	0.18519828	0.19422475
TRBV12-4_TRBJ2-4	0.78247022	1.13E-10	1.46E-10
TRBV12-4_TRBJ2-5	1.27048053	0.02220703	0.02418357
TRBV12-4_TRBJ2-6	1.20845902	0.08013267	0.08606332
TRBV12-4_TRBJ2-7	0.83458144	4.52E-23	6.88E-23
TRBV12-5_TRBJ1-1	1.0197991	1.15E-33	1.94E-33
TRBV12-5_TRBJ1-2	1.70177837	5.32E-30	8.71E-30
TRBV12-5_TRBJ1-3	0.09665887	5.30E-126	1.77E-125
TRBV12-5_TRBJ1-4	1.09238985	0.00246715	0.00279401
TRBV12-5_TRBJ1-5	1.05142846	1.71E-07	2.12E-07
TRBV12-5_TRBJ1-6	3.28032412	2.47E-05	2.92E-05
TRBV12-5_TRBJ2-1	1.00608807	2.22E-51	4.40E-51
TRBV12-5_TRBJ2-2	2.05173757	0.55129816	0.56302791
TRBV12-5_TRBJ2-3	1.9639961	2.08E-29	3.38E-29
TRBV12-5_TRBJ2-4	0.42351595	0.01112069	0.01228196
TRBV12-5_TRBJ2-5	0.98142433	2.04E-08	2.56E-08
TRBV12-5_TRBJ2-6	1.11276437	0.00202302	0.00230359
TRBV12-5_TRBJ2-7	0.58797737	6.81E-24	1.04E-23
TRBV13_TRBJ1-1	0.86149377	4.39E-79	1.08E-78
TRBV13_TRBJ1-2	1.93368412	1.59E-06	1.92E-06
TRBV13_TRBJ1-3	0.0817584	1.30E-170	7.87E-170
TRBV13_TRBJ1-4	1.3464083	0.0924495	0.09878166
TRBV13_TRBJ1-5	0.93059779	8.52E-05	9.98E-05
TRBV13_TRBJ1-6	4.08621155	1.24E-146	5.14E-146
TRBV13_TRBJ2-1	0.8963287	8.81E-73	2.01E-72

TRBV13_TRBJ2-2	1.5593276	2.27E-26	3.55E-26
TRBV13_TRBJ2-3	0.82608939	5.98E-48	1.15E-47
TRBV13_TRBJ2-4	1.2013305	1.60E-15	2.24E-15
TRBV13_TRBJ2-5	0.56617885	6.25E-96	1.74E-95
TRBV13_TRBJ2-6	0.49847083	7.75E-13	1.04E-12
TRBV13_TRBJ2-7	0.77784098	6.53E-77	1.56E-76
TRBV14_TRBJ1-1	0.21956471	2.66E-157	1.29E-156
TRBV14_TRBJ1-2	1.2435468	6.57E-08	8.18E-08
TRBV14_TRBJ1-3	0.05483141	9.66E-221	1.51E-218
TRBV14_TRBJ1-4	0.62906601	5.65E-36	9.77E-36
TRBV14_TRBJ1-5	0.40019698	6.38E-54	1.30E-53
TRBV14_TRBJ1-6	2.6483578	1.87E-75	4.33E-75
TRBV14_TRBJ2-1	0.44446295	2.26E-157	1.11E-156
TRBV14_TRBJ2-2	0.48483324	1.17E-98	3.29E-98
TRBV14_TRBJ2-3	0.32310549	8.73E-143	3.40E-142
TRBV14_TRBJ2-4	0.16720066	1.52E-14	2.08E-14
TRBV14_TRBJ2-5	0.30994405	1.29E-160	6.63E-160
TRBV14_TRBJ2-6	0.45793263	3.17E-44	5.90E-44
TRBV14_TRBJ2-7	0.27210052	5.56E-168	3.15E-167
TRBV15_TRBJ1-1	1.36026148	0.0075667	0.00841644
TRBV15_TRBJ1-2	2.03790761	2.56E-101	7.36E-101
TRBV15_TRBJ1-3	0.3529687	8.39E-173	5.34E-172
TRBV15_TRBJ1-4	2.41318702	1.14E-51	2.28E-51
TRBV15_TRBJ1-5	1.66566899	3.05E-33	5.09E-33
TRBV15_TRBJ1-6	6.95182127	4.86E-136	1.77E-135
TRBV15_TRBJ2-1	1.39618174	8.93E-08	1.11E-07
TRBV15_TRBJ2-2	1.21507498	0.00497781	0.00557657
TRBV15_TRBJ2-3	1.34637805	5.78E-11	7.56E-11
TRBV15_TRBJ2-4	0.42406659	0.151663	0.159861
TRBV15_TRBJ2-5	1.7082739	0.00441959	0.00496906
TRBV15_TRBJ2-6	1.05812036	3.41E-08	4.26E-08
TRBV15_TRBJ2-7	1.14167908	1.92E-15	2.68E-15
TRBV16_TRBJ1-1	1.48658255	0.07411759	0.0797403
TRBV16_TRBJ1-2	3.45884053	2.41E-29	3.89E-29
TRBV16_TRBJ1-3	0.05766515	1.22E-20	1.83E-20
TRBV16_TRBJ1-4	1.06253046	3.25E-16	4.58E-16
TRBV16_TRBJ1-5	1.15303864	0.00111264	0.00127392
TRBV16_TRBJ1-6	7.55848028	1.47E-86	3.83E-86
TRBV16_TRBJ2-1	0.78507696	0.50966941	0.52222285
TRBV16_TRBJ2-2	1.53614949	1.02E-08	1.28E-08
TRBV16_TRBJ2-3	2.44946482	4.26E-17	6.08E-17
TRBV16_TRBJ2-4	0.27317052	7.12E-07	8.75E-07

TRBV16_TRBJ2-5	0.4399007	3.45E-07	4.25E-07
TRBV16_TRBJ2-6	0.92050641	3.36E-42	6.09E-42
TRBV16_TRBJ2-7	1.23924369	0.00012948	0.00015074
TRBV18_TRBJ1-1	1.13703383	0.14468832	0.15276736
TRBV18_TRBJ1-2	1.08902755	0.12646078	0.13374835
TRBV18_TRBJ1-3	0.12483964	2.08E-168	1.20E-167
TRBV18_TRBJ1-4	2.86119293	2.00E-33	3.35E-33
TRBV18_TRBJ1-5	1.06652291	2.02E-05	2.40E-05
TRBV18_TRBJ1-6	0.86601565	6.61E-33	1.10E-32
TRBV18_TRBJ2-1	0.55839663	1.15E-47	2.19E-47
TRBV18_TRBJ2-2	1.42069475	2.73E-09	3.46E-09
TRBV18_TRBJ2-3	0.75787758	4.37E-23	6.66E-23
TRBV18_TRBJ2-4	0.38916137	1.71E-60	3.64E-60
TRBV18_TRBJ2-5	0.63423874	4.43E-91	1.18E-90
TRBV18_TRBJ2-6	0.88333592	1.08E-06	1.32E-06
TRBV18_TRBJ2-7	0.70817929	6.36E-31	1.06E-30
TRBV19_TRBJ1-1	0.69457088	2.41E-59	5.07E-59
TRBV19_TRBJ1-2	2.12062414	2.64E-77	6.33E-77
TRBV19_TRBJ1-3	0.05291716	3.22E-209	2.51E-207
TRBV19_TRBJ1-4	1.95888994	1.75E-25	2.71E-25
TRBV19_TRBJ1-5	1.25694465	0.94308974	0.94460353
TRBV19_TRBJ1-6	3.66891843	6.40E-159	3.22E-158
TRBV19_TRBJ2-1	1.08683824	1.77E-16	2.51E-16
TRBV19_TRBJ2-2	1.06382298	0.27447116	0.28545
TRBV19_TRBJ2-3	0.7075623	1.90E-34	3.23E-34
TRBV19_TRBJ2-4	0.51111942	5.06E-49	9.80E-49
TRBV19_TRBJ2-5	0.86966588	3.35E-28	5.32E-28
TRBV19_TRBJ2-6	0.85807834	8.63E-21	1.29E-20
TRBV19_TRBJ2-7	0.58269682	1.98E-75	4.58E-75
TRBV2_TRBJ1-1	0.85989825	6.34E-29	1.02E-28
TRBV2_TRBJ1-2	1.63879187	1.81E-43	3.36E-43
TRBV2_TRBJ1-3	0.08227672	1.10E-188	1.19E-187
TRBV2_TRBJ1-4	1.47741311	4.59E-28	7.25E-28
TRBV2_TRBJ1-5	1.26366589	0.78345776	0.78978617
TRBV2_TRBJ1-6	6.01886513	7.85E-179	5.83E-178
TRBV2_TRBJ2-1	0.75314593	3.98E-60	8.45E-60
TRBV2_TRBJ2-2	0.97170384	0.16596198	0.17463791
TRBV2_TRBJ2-3	0.86697944	1.51E-18	2.19E-18
TRBV2_TRBJ2-4	0.38420736	2.45E-73	5.61E-73
TRBV2_TRBJ2-5	1.05320452	2.52E-11	3.31E-11
TRBV2_TRBJ2-6	1.16558072	2.69E-23	4.12E-23
TRBV2_TRBJ2-7	0.65666307	1.51E-63	3.28E-63

TRBV20-1_TRBJ1-1	0.33789523	1.78E-180	1.40E-179
TRBV20-1_TRBJ1-2	0.68150307	6.82E-76	1.59E-75
TRBV20-1_TRBJ1-3	0.04871193	1.96E-198	4.71E-197
TRBV20-1_TRBJ1-4	0.8813925	3.29E-06	3.95E-06
TRBV20-1_TRBJ1-5	0.7463484	7.30E-31	1.21E-30
TRBV20-1_TRBJ1-6	0.88872598	3.10E-38	5.48E-38
TRBV20-1_TRBJ2-1	0.44016671	1.72E-149	7.45E-149
TRBV20-1_TRBJ2-2	0.32874284	2.95E-173	1.90E-172
TRBV20-1_TRBJ2-3	0.74397301	3.39E-106	9.94E-106
TRBV20-1_TRBJ2-4	0.25541019	2.57E-155	1.22E-154
TRBV20-1_TRBJ2-5	0.41319727	1.09E-165	5.95E-165
TRBV20-1_TRBJ2-6	0.4682901	1.08E-135	3.91E-135
TRBV20-1_TRBJ2-7	0.52366203	4.39E-132	1.56E-131
TRBV24-1_TRBJ1-1	0.674213	1.07E-30	1.77E-30
TRBV24-1_TRBJ1-2	2.18281862	1.73E-71	3.91E-71
TRBV24-1_TRBJ1-3	0.03715171	1.02E-152	4.66E-152
TRBV24-1_TRBJ1-4	1.8423584	1.84E-12	2.45E-12
TRBV24-1_TRBJ1-5	1.53539234	0.11809287	0.12511028
TRBV24-1_TRBJ1-6	3.39145737	6.10E-28	9.61E-28
TRBV24-1_TRBJ2-1	1.2919545	1.43E-17	2.06E-17
TRBV24-1_TRBJ2-2	0.80889886	1.02E-48	1.97E-48
TRBV24-1_TRBJ2-3	0.93837823	2.00E-06	2.40E-06
TRBV24-1_TRBJ2-4	0.64716151	0.0093423	0.01037294
TRBV24-1_TRBJ2-5	0.84806411	5.05E-09	6.40E-09
TRBV24-1_TRBJ2-6	1.65976995	6.87E-05	8.07E-05
TRBV24-1_TRBJ2-7	0.57535128	1.97E-52	3.99E-52
TRBV25-1_TRBJ1-1	0.75723165	1.49E-16	2.12E-16
TRBV25-1_TRBJ1-2	2.35859609	2.61E-70	5.89E-70
TRBV25-1_TRBJ1-3	0.0355179	5.28E-189	5.78E-188
TRBV25-1_TRBJ1-4	0.74138414	0.00113582	0.00129808
TRBV25-1_TRBJ1-5	0.86734583	1.10E-13	1.49E-13
TRBV25-1_TRBJ1-6	30.0664444	6.49E-196	1.02E-194
TRBV25-1_TRBJ2-1	0.59179181	1.68E-65	3.69E-65
TRBV25-1_TRBJ2-2	0.89882574	1.05E-18	1.53E-18
TRBV25-1_TRBJ2-3	0.36708947	6.13E-77	1.47E-76
TRBV25-1_TRBJ2-4	0.239995	0.00011902	0.00013882
TRBV25-1_TRBJ2-5	0.81723987	0.00181494	0.00207042
TRBV25-1_TRBJ2-6	0.51163773	1.25E-33	2.10E-33
TRBV25-1_TRBJ2-7	0.74965764	2.39E-76	5.64E-76
TRBV27_TRBJ1-1	1.13829669	0.40850656	0.42064042
TRBV27_TRBJ1-2	2.35175227	2.42E-85	6.22E-85
TRBV27_TRBJ1-3	0.17027262	1.56E-179	1.17E-178

TRBV27_TRBJ1-4	1.81535356	5.71E-43	1.05E-42
TRBV27_TRBJ1-5	1.57249397	7.06E-11	9.22E-11
TRBV27_TRBJ1-6	4.13836333	1.86E-119	5.90E-119
TRBV27_TRBJ2-1	0.92981783	4.25E-37	7.43E-37
TRBV27_TRBJ2-2	1.12327503	0.02711817	0.02942911
TRBV27_TRBJ2-3	1.25251581	0.33129221	0.34282975
TRBV27_TRBJ2-4	0.72764463	1.26E-09	1.60E-09
TRBV27_TRBJ2-5	0.88310494	7.85E-09	9.92E-09
TRBV27_TRBJ2-6	1.2183381	0.013042	0.0143531
TRBV27_TRBJ2-7	0.98502567	1.32E-19	1.93E-19
TRBV28_TRBJ1-1	0.37849713	2.97E-131	1.03E-130
TRBV28_TRBJ1-2	0.76485269	3.04E-34	5.15E-34
TRBV28_TRBJ1-3	0.05835051	1.45E-196	2.50E-195
TRBV28_TRBJ1-4	0.42765592	1.59E-98	4.45E-98
TRBV28_TRBJ1-5	0.54059205	7.94E-123	2.58E-122
TRBV28_TRBJ1-6	1.77665107	0.0422516	0.04569324
TRBV28_TRBJ2-1	0.26918863	9.73E-153	4.46E-152
TRBV28_TRBJ2-2	0.40215607	1.26E-113	3.85E-113
TRBV28_TRBJ2-3	0.34957246	6.32E-136	2.29E-135
TRBV28_TRBJ2-4	0.13982038	1.11E-138	4.25E-138
TRBV28_TRBJ2-5	0.25333332	1.47E-137	5.53E-137
TRBV28_TRBJ2-6	0.19436257	6.74E-150	2.94E-149
TRBV28_TRBJ2-7	0.31034137	5.32E-147	2.23E-146
TRBV29-1_TRBJ1-1	0.51300845	7.81E-104	2.27E-103
TRBV29-1_TRBJ1-2	1.05257691	0.06827715	0.07358366
TRBV29-1_TRBJ1-3	0.05043613	3.08E-235	1.92E-232
TRBV29-1_TRBJ1-4	1.00527264	1.89E-12	2.51E-12
TRBV29-1_TRBJ1-5	0.60167929	8.73E-42	1.58E-41
TRBV29-1_TRBJ1-6	1.62230168	9.08E-25	1.40E-24
TRBV29-1_TRBJ2-1	0.4841064	7.33E-118	2.29E-117
TRBV29-1_TRBJ2-2	0.82773792	1.48E-51	2.95E-51
TRBV29-1_TRBJ2-3	0.32705975	7.93E-144	3.13E-143
TRBV29-1_TRBJ2-4	0.1667875	1.15E-10	1.48E-10
TRBV29-1_TRBJ2-5	0.79255712	3.59E-51	7.09E-51
TRBV29-1_TRBJ2-6	0.42323655	6.08E-73	1.39E-72
TRBV29-1_TRBJ2-7	0.36609408	9.78E-138	3.70E-137
TRBV30_TRBJ1-1	1.49491002	1.25E-06	1.53E-06
TRBV30_TRBJ1-2	1.62104638	3.32E-17	4.75E-17
TRBV30_TRBJ1-3	0.20755308	7.89E-145	3.15E-144
TRBV30_TRBJ1-4	2.5727476	1.02E-72	2.32E-72
TRBV30_TRBJ1-5	1.55878835	4.44E-14	6.04E-14
TRBV30_TRBJ1-6	1.57582432	4.55E-07	5.60E-07

TRBV30_TRBJ2-1	0.84002339	3.00E-16	4.24E-16
TRBV30_TRBJ2-2	1.21073964	0.00207559	0.00235485
TRBV30_TRBJ2-3	1.02928486	0.00520501	0.00581024
TRBV30_TRBJ2-4	0.96550667	1.28E-09	1.63E-09
TRBV30_TRBJ2-5	0.87639808	1.80E-17	2.58E-17
TRBV30_TRBJ2-6	1.78222564	0.54392154	0.55640498
TRBV30_TRBJ2-7	1.12847164	0.90723103	0.91014817
TRBV3-1_TRBJ1-1	0.14689203	1.20E-189	1.36E-188
TRBV3-1_TRBJ1-2	0.20434686	4.96E-182	4.02E-181
TRBV3-1_TRBJ1-3	0.00592674	1.38E-220	1.72E-218
TRBV3-1_TRBJ1-4	0.29816409	5.52E-131	1.92E-130
TRBV3-1_TRBJ1-5	0.17947779	3.78E-175	2.54E-174
TRBV3-1_TRBJ1-6	0.49213279	2.65E-113	8.04E-113
TRBV3-1_TRBJ2-1	0.13207336	5.49E-188	5.62E-187
TRBV3-1_TRBJ2-2	0.13213548	7.34E-186	6.74E-185
TRBV3-1_TRBJ2-3	0.31121055	1.47E-188	1.56E-187
TRBV3-1_TRBJ2-4	0.05455647	7.96E-151	3.52E-150
TRBV3-1_TRBJ2-5	0.09307787	1.16E-194	1.69E-193
TRBV3-1_TRBJ2-6	0.12179662	4.75E-177	3.40E-176
TRBV3-1_TRBJ2-7	0.10887487	6.86E-195	1.02E-193
TRBV4-1_TRBJ1-1	0.28346589	2.37E-149	1.02E-148
TRBV4-1_TRBJ1-2	0.26538358	2.24E-163	1.20E-162
TRBV4-1_TRBJ1-3	0.04942969	3.96E-209	2.74E-207
TRBV4-1_TRBJ1-4	1.04829267	1.36E-13	1.84E-13
TRBV4-1_TRBJ1-5	0.50090235	3.93E-90	1.04E-89
TRBV4-1_TRBJ1-6	0.45972416	1.00E-93	2.74E-93
TRBV4-1_TRBJ2-1	0.29657023	1.80E-168	1.05E-167
TRBV4-1_TRBJ2-2	0.42526246	2.52E-160	1.29E-159
TRBV4-1_TRBJ2-3	0.36919416	8.52E-146	3.45E-145
TRBV4-1_TRBJ2-4	0.3775373	3.15E-84	8.05E-84
TRBV4-1_TRBJ2-5	0.25333935	1.36E-171	8.46E-171
TRBV4-1_TRBJ2-6	0.27230835	1.37E-136	5.05E-136
TRBV4-1_TRBJ2-7	0.33117722	2.69E-157	1.30E-156
TRBV4-2_TRBJ1-1	0.46583045	3.20E-117	9.88E-117
TRBV4-2_TRBJ1-2	0.78156156	8.86E-28	1.39E-27
TRBV4-2_TRBJ1-3	0.06394969	3.17E-187	3.14E-186
TRBV4-2_TRBJ1-4	2.04983085	4.60E-06	5.51E-06
TRBV4-2_TRBJ1-5	0.55587104	4.29E-50	8.37E-50
TRBV4-2_TRBJ1-6	0.8760778	1.26E-11	1.66E-11
TRBV4-2_TRBJ2-1	0.3742354	7.02E-144	2.79E-143
TRBV4-2_TRBJ2-2	0.46693322	1.85E-104	5.39E-104
TRBV4-2_TRBJ2-3	0.46279466	3.88E-123	1.27E-122

TRBV4-2_TRBJ2-4	0.31196895	9.56E-30	1.56E-29
TRBV4-2_TRBJ2-5	0.25160294	4.76E-160	2.42E-159
TRBV4-2_TRBJ2-6	0.23885439	9.91E-110	2.97E-109
TRBV4-2_TRBJ2-7	0.34797348	1.37E-146	5.64E-146
TRBV4-3_TRBJ1-1	0.4607684	0.00944192	0.01046493
TRBV4-3_TRBJ1-2	0.65465988	0.02199181	0.02399107
TRBV4-3_TRBJ1-3	0.15941936	2.73E-62	5.86E-62
TRBV4-3_TRBJ1-4	1.17377964	0.00424191	0.00477789
TRBV4-3_TRBJ1-5	0.54594666	0.86034124	0.86449747
TRBV4-3_TRBJ1-6	0.54980579	0.95508825	0.95508825
TRBV4-3_TRBJ2-1	0.30362888	3.04E-05	3.58E-05
TRBV4-3_TRBJ2-2	0.59454337	0.08533276	0.09133386
TRBV4-3_TRBJ2-3	0.32035903	0.00048627	0.00055881
TRBV4-3_TRBJ2-4	0.52928882	0.01383587	0.01519997
TRBV4-3_TRBJ2-5	0.38095947	2.60E-05	3.07E-05
TRBV4-3_TRBJ2-6	0.23928568	0.00017834	0.00020723
TRBV4-3_TRBJ2-7	0.50328362	0.01299765	0.01432956
TRBV5-1_TRBJ1-1	1.18471618	0.00508808	0.00568989
TRBV5-1_TRBJ1-2	1.59413314	9.00E-26	1.40E-25
TRBV5-1_TRBJ1-3	0.07549775	3.82E-185	3.36E-184
TRBV5-1_TRBJ1-4	2.06543358	2.53E-43	4.68E-43
TRBV5-1_TRBJ1-5	1.0696754	0.00341861	0.00385753
TRBV5-1_TRBJ1-6	2.20737539	4.71E-34	7.97E-34
TRBV5-1_TRBJ2-1	1.25734864	0.69671762	0.70691349
TRBV5-1_TRBJ2-2	1.34913117	0.01566177	0.01717565
TRBV5-1_TRBJ2-3	1.16973147	0.09712895	0.10360422
TRBV5-1_TRBJ2-4	0.62223784	1.34E-28	2.13E-28
TRBV5-1_TRBJ2-5	0.71308966	3.42E-45	6.48E-45
TRBV5-1_TRBJ2-6	0.85959668	5.32E-16	7.48E-16
TRBV5-1_TRBJ2-7	0.86713736	4.22E-13	5.66E-13
TRBV5-4_TRBJ1-1	1.87635864	4.72E-26	7.36E-26
TRBV5-4_TRBJ1-2	2.06938072	5.67E-51	1.12E-50
TRBV5-4_TRBJ1-3	0.20253114	1.97E-133	7.04E-133
TRBV5-4_TRBJ1-4	5.20092489	2.09E-86	5.43E-86
TRBV5-4_TRBJ1-5	1.18181403	4.16E-55	8.53E-55
TRBV5-4_TRBJ1-6	7.41537738	7.11E-132	2.51E-131
TRBV5-4_TRBJ2-1	0.91619717	0.63632461	0.64774316
TRBV5-4_TRBJ2-2	3.29758547	3.38E-66	7.51E-66
TRBV5-4_TRBJ2-3	2.00163595	1.26E-16	1.79E-16
TRBV5-4_TRBJ2-4	1.04937794	2.40E-20	3.58E-20
TRBV5-4_TRBJ2-5	1.23955652	0.080324	0.08612058
TRBV5-4_TRBJ2-6	1.32665154	7.58E-12	1.00E-11

TRBV5-4_TRBJ2-7	1.34669104	0.00039254	0.00045193
TRBV5-5_TRBJ1-1	0.3126517	3.57E-148	1.52E-147
TRBV5-5_TRBJ1-2	0.91217184	0.00586154	0.00653143
TRBV5-5_TRBJ1-3	0.0681526	1.45E-187	1.46E-186
TRBV5-5_TRBJ1-4	0.69183546	6.29E-57	1.30E-56
TRBV5-5_TRBJ1-5	0.42608248	5.23E-92	1.41E-91
TRBV5-5_TRBJ1-6	1.8296494	2.44E-37	4.29E-37
TRBV5-5_TRBJ2-1	0.40650571	6.44E-120	2.05E-119
TRBV5-5_TRBJ2-2	0.65636324	2.35E-65	5.17E-65
TRBV5-5_TRBJ2-3	0.65340324	8.74E-83	2.20E-82
TRBV5-5_TRBJ2-4	0.16232021	5.11E-10	6.55E-10
TRBV5-5_TRBJ2-5	0.22826119	7.23E-162	3.82E-161
TRBV5-5_TRBJ2-6	0.42896072	1.16E-44	2.19E-44
TRBV5-5_TRBJ2-7	0.3150514	1.42E-158	7.11E-158
TRBV5-6_TRBJ1-1	0.13223997	2.64E-185	2.36E-184
TRBV5-6_TRBJ1-2	0.17024455	1.28E-179	9.71E-179
TRBV5-6_TRBJ1-3	0.02023041	5.93E-232	1.85E-229
TRBV5-6_TRBJ1-4	0.31957889	3.36E-130	1.15E-129
TRBV5-6_TRBJ1-5	0.16941445	2.58E-174	1.68E-173
TRBV5-6_TRBJ1-6	0.15203145	3.05E-176	2.12E-175
TRBV5-6_TRBJ2-1	0.10469047	8.01E-190	9.26E-189
TRBV5-6_TRBJ2-2	0.12849449	1.71E-176	1.20E-175
TRBV5-6_TRBJ2-3	0.1326758	7.04E-186	6.66E-185
TRBV5-6_TRBJ2-4	0.04844007	1.75E-49	3.41E-49
TRBV5-6_TRBJ2-5	0.08362502	6.70E-183	5.50E-182
TRBV5-6_TRBJ2-6	0.11444566	5.29E-152	2.38E-151
TRBV5-6_TRBJ2-7	0.14855274	9.59E-180	7.39E-179
TRBV5-8_TRBJ1-1	0.45220034	4.70E-130	1.60E-129
TRBV5-8_TRBJ1-2	0.80420908	4.79E-55	9.80E-55
TRBV5-8_TRBJ1-3	0.06310465	2.37E-189	2.64E-188
TRBV5-8_TRBJ1-4	1.04111554	0.01892284	0.02067925
TRBV5-8_TRBJ1-5	0.86735186	1.72E-35	2.97E-35
TRBV5-8_TRBJ1-6	0.56035062	3.79E-109	1.13E-108
TRBV5-8_TRBJ2-1	0.25463762	5.74E-170	3.41E-169
TRBV5-8_TRBJ2-2	0.58146069	2.60E-80	6.43E-80
TRBV5-8_TRBJ2-3	0.4702622	6.46E-140	2.49E-139
TRBV5-8_TRBJ2-4	0.26870615	7.86E-11	1.02E-10
TRBV5-8_TRBJ2-5	0.29335705	3.01E-153	1.40E-152
TRBV5-8_TRBJ2-6	0.30058394	2.18E-83	5.54E-83
TRBV5-8_TRBJ2-7	0.40253032	5.97E-137	2.22E-136
TRBV6-1_TRBJ1-1	1.26604564	0.67776667	0.68880522
TRBV6-1_TRBJ1-2	2.95540859	2.44E-131	8.55E-131

TRBV6-1_TRBJ1-3	0.11823533	1.76E-174	1.15E-173
TRBV6-1_TRBJ1-4	2.3839408	2.53E-61	5.41E-61
TRBV6-1_TRBJ1-5	1.50763015	9.43E-22	1.42E-21
TRBV6-1_TRBJ1-6	2.01781198	6.58E-49	1.27E-48
TRBV6-1_TRBJ2-1	0.72044726	3.33E-57	6.91E-57
TRBV6-1_TRBJ2-2	1.10444369	1.47E-06	1.78E-06
TRBV6-1_TRBJ2-3	0.82730773	5.13E-20	7.57E-20
TRBV6-1_TRBJ2-4	1.32455776	2.31E-15	3.21E-15
TRBV6-1_TRBJ2-5	1.2158792	0.05699439	0.06153027
TRBV6-1_TRBJ2-6	0.72274457	1.70E-34	2.90E-34
TRBV6-1_TRBJ2-7	0.88565592	1.03E-22	1.56E-22
TRBV6-2_TRBJ1-1	0.75985905	2.78E-58	5.83E-58
TRBV6-2_TRBJ1-2	2.0982247	4.97E-30	8.15E-30
TRBV6-2_TRBJ1-3	0.04688723	3.27E-197	6.18E-196
TRBV6-2_TRBJ1-4	0.62465841	5.67E-90	1.49E-89
TRBV6-2_TRBJ1-5	1.21475044	9.10E-09	1.15E-08
TRBV6-2_TRBJ1-6	2.27155314	1.70E-51	3.38E-51
TRBV6-2_TRBJ2-1	0.62038562	5.58E-86	1.45E-85
TRBV6-2_TRBJ2-2	0.87087376	3.06E-58	6.39E-58
TRBV6-2_TRBJ2-3	0.62274722	1.75E-77	4.24E-77
TRBV6-2_TRBJ2-4	0.62366685	1.94E-40	3.47E-40
TRBV6-2_TRBJ2-5	0.78002054	2.14E-44	4.01E-44
TRBV6-2_TRBJ2-6	0.75822182	2.00E-50	3.91E-50
TRBV6-2_TRBJ2-7	0.62026916	4.12E-76	9.65E-76
TRBV6-3_TRBJ1-1	0.76031116	3.40E-58	7.08E-58
TRBV6-3_TRBJ1-2	2.09698626	5.46E-30	8.92E-30
TRBV6-3_TRBJ1-3	0.04708352	2.88E-197	5.79E-196
TRBV6-3_TRBJ1-4	0.62836504	9.83E-90	2.58E-89
TRBV6-3_TRBJ1-5	1.21683994	9.14E-09	1.15E-08
TRBV6-3_TRBJ1-6	2.27767593	4.23E-52	8.49E-52
TRBV6-3_TRBJ2-1	0.62069908	1.70E-85	4.39E-85
TRBV6-3_TRBJ2-2	0.87106964	2.07E-58	4.35E-58
TRBV6-3_TRBJ2-3	0.62198732	1.14E-77	2.79E-77
TRBV6-3_TRBJ2-4	0.62154824	1.59E-39	2.82E-39
TRBV6-3_TRBJ2-5	0.7776749	1.15E-44	2.17E-44
TRBV6-3_TRBJ2-6	0.75836942	8.81E-51	1.73E-50
TRBV6-3_TRBJ2-7	0.62034643	3.33E-76	7.83E-76
TRBV6-4_TRBJ1-1	1.29886611	5.84E-06	6.98E-06
TRBV6-4_TRBJ1-2	5.55838286	4.53E-185	3.93E-184
TRBV6-4_TRBJ1-3	0.16360834	6.05E-152	2.70E-151
TRBV6-4_TRBJ1-4	2.30874374	2.25E-59	4.77E-59
TRBV6-4_TRBJ1-5	1.54250434	2.81E-26	4.39E-26

TRBV6-4_TRBJ1-6	13.8265771	2.32E-192	3.01E-191
TRBV6-4_TRBJ2-1	0.98953458	1.21E-09	1.54E-09
TRBV6-4_TRBJ2-2	0.89689716	1.82E-14	2.49E-14
TRBV6-4_TRBJ2-3	0.60939288	3.93E-37	6.88E-37
TRBV6-4_TRBJ2-4	1.69697375	1.42E-21	2.14E-21
TRBV6-4_TRBJ2-5	1.43477738	0.00077659	0.0008908
TRBV6-4_TRBJ2-6	0.6889487	7.39E-11	9.62E-11
TRBV6-4_TRBJ2-7	1.01161502	0.10164798	0.10823949
TRBV6-5_TRBJ1-1	1.9854441	4.57E-38	8.05E-38
TRBV6-5_TRBJ1-2	3.84123525	1.22E-163	6.59E-163
TRBV6-5_TRBJ1-3	0.1897018	4.36E-161	2.28E-160
TRBV6-5_TRBJ1-4	4.28716243	4.84E-149	2.07E-148
TRBV6-5_TRBJ1-5	1.78519912	1.19E-42	2.17E-42
TRBV6-5_TRBJ1-6	5.13539432	7.86E-166	4.34E-165
TRBV6-5_TRBJ2-1	1.26088926	0.39482641	0.40722591
TRBV6-5_TRBJ2-2	1.55196144	1.65E-11	2.17E-11
TRBV6-5_TRBJ2-3	1.26742016	1.20E-09	1.53E-09
TRBV6-5_TRBJ2-4	1.76908614	8.28E-43	1.51E-42
TRBV6-5_TRBJ2-5	1.64921945	3.77E-28	5.98E-28
TRBV6-5_TRBJ2-6	1.68246697	0.24374996	0.25434778
TRBV6-5_TRBJ2-7	1.21366725	0.11303251	0.12015722
TRBV6-6_TRBJ1-1	26.3509702	1.20E-199	4.50E-198
TRBV6-6_TRBJ1-2	76.1607187	1.57E-200	8.26E-199
TRBV6-6_TRBJ1-3	3.13034117	1.98E-14	2.71E-14
TRBV6-6_TRBJ1-4	17.7141721	1.53E-192	2.03E-191
TRBV6-6_TRBJ1-5	34.9097286	7.28E-199	2.16E-197
TRBV6-6_TRBJ1-6	89.5479956	1.72E-200	8.26E-199
TRBV6-6_TRBJ2-1	17.9317014	2.72E-196	4.47E-195
TRBV6-6_TRBJ2-2	28.2873611	1.32E-198	3.59E-197
TRBV6-6_TRBJ2-3	21.9391603	8.26E-198	1.84E-196
TRBV6-6_TRBJ2-4	18.4787858	4.24E-171	2.59E-170
TRBV6-6_TRBJ2-5	14.4540247	1.26E-184	1.06E-183
TRBV6-6_TRBJ2-6	26.2619689	1.70E-188	1.77E-187
TRBV6-6_TRBJ2-7	19.9756856	5.55E-199	1.81E-197
TRBV6-8_TRBJ1-1	0.03019026	1.14E-199	4.50E-198
TRBV6-8_TRBJ1-2	0.10223618	3.90E-192	4.87E-191
TRBV6-8_TRBJ1-3	0.00238126	1.54E-218	1.60E-216
TRBV6-8_TRBJ1-4	0.10673379	2.81E-191	3.44E-190
TRBV6-8_TRBJ1-5	0.05427428	3.38E-197	6.20E-196
TRBV6-8_TRBJ1-6	0.32964476	2.08E-120	6.64E-120
TRBV6-8_TRBJ2-1	0.04249833	4.49E-199	1.56E-197
TRBV6-8_TRBJ2-2	0.03358244	1.23E-198	3.49E-197

TRBV6-8_TRBJ2-3	0.0402469	5.81E-199	1.81E-197
TRBV6-8_TRBJ2-4	0.10836512	2.74E-119	8.63E-119
TRBV6-8_TRBJ2-5	0.10303662	2.29E-195	3.49E-194
TRBV6-8_TRBJ2-6	0.03262061	1.29E-197	2.67E-196
TRBV6-8_TRBJ2-7	0.02476107	1.70E-200	8.26E-199
TRBV6-9_TRBJ1-1	0.14360123	4.31E-191	5.18E-190
TRBV6-9_TRBJ1-2	0.20521319	4.25E-164	2.30E-163
TRBV6-9_TRBJ1-3	0.01260544	5.48E-216	4.88E-214
TRBV6-9_TRBJ1-4	0.95263733	2.34E-10	3.02E-10
TRBV6-9_TRBJ1-5	0.16687436	3.49E-181	2.80E-180
TRBV6-9_TRBJ1-6	1.86469607	1.91E-08	2.39E-08
TRBV6-9_TRBJ2-1	0.11411053	2.31E-194	3.27E-193
TRBV6-9_TRBJ2-2	0.1042437	1.13E-192	1.53E-191
TRBV6-9_TRBJ2-3	0.09354661	3.05E-197	5.94E-196
TRBV6-9_TRBJ2-4	0.49048465	6.73E-25	1.04E-24
TRBV6-9_TRBJ2-5	0.34213239	1.76E-122	5.70E-122
TRBV6-9_TRBJ2-6	0.07625447	6.19E-185	5.29E-184
TRBV6-9_TRBJ2-7	0.12527808	2.85E-190	3.35E-189
TRBV7-2_TRBJ1-1	0.77401929	2.75E-20	4.09E-20
TRBV7-2_TRBJ1-2	1.79141495	9.41E-14	1.28E-13
TRBV7-2_TRBJ1-3	0.11537819	3.43E-171	2.12E-170
TRBV7-2_TRBJ1-4	1.21935722	0.02262278	0.02459341
TRBV7-2_TRBJ1-5	0.75415223	4.84E-20	7.16E-20
TRBV7-2_TRBJ1-6	1.19136282	1.60E-06	1.94E-06
TRBV7-2_TRBJ2-1	0.55436345	2.33E-52	4.70E-52
TRBV7-2_TRBJ2-2	1.10306686	0.00445819	0.00500344
TRBV7-2_TRBJ2-3	0.8102228	4.97E-15	6.85E-15
TRBV7-2_TRBJ2-4	0.41750103	2.13E-47	4.05E-47
TRBV7-2_TRBJ2-5	0.61205312	3.54E-40	6.33E-40
TRBV7-2_TRBJ2-6	0.76354845	2.22E-14	3.02E-14
TRBV7-2_TRBJ2-7	0.63902673	1.07E-41	1.94E-41
TRBV7-3_TRBJ1-1	0.89927743	2.89E-13	3.88E-13
TRBV7-3_TRBJ1-2	2.2959527	4.30E-66	9.52E-66
TRBV7-3_TRBJ1-3	0.12566432	1.73E-147	7.29E-147
TRBV7-3_TRBJ1-4	1.4790107	7.94E-20	1.17E-19
TRBV7-3_TRBJ1-5	0.77955331	0.00027613	0.00031967
TRBV7-3_TRBJ1-6	4.3214902	5.54E-64	1.20E-63
TRBV7-3_TRBJ2-1	0.9954913	2.40E-29	3.89E-29
TRBV7-3_TRBJ2-2	1.33378475	1.96E-35	3.37E-35
TRBV7-3_TRBJ2-3	0.9001725	6.31E-05	7.43E-05
TRBV7-3_TRBJ2-4	0.59375258	0.29659768	0.30794834
TRBV7-3_TRBJ2-5	0.85517384	2.32E-15	3.21E-15

TRBV7-3_TRBJ2-6	0.75464247	1.30E-07	1.62E-07
TRBV7-3_TRBJ2-7	0.84366971	1.45E-19	2.12E-19
TRBV7-4_TRBJ1-1	1.46725022	9.36E-69	2.10E-68
TRBV7-4_TRBJ1-2	7.01597757	2.92E-143	1.15E-142
TRBV7-4_TRBJ1-3	0.1524422	0.56016776	0.57115144
TRBV7-4_TRBJ1-4	0.87729498	2.64E-78	6.47E-78
TRBV7-4_TRBJ1-5	0.78337728	3.70E-43	6.81E-43
TRBV7-4_TRBJ1-6	2.19556542	6.47E-127	2.17E-126
TRBV7-4_TRBJ2-1	3.77027133	2.09E-106	6.14E-106
TRBV7-4_TRBJ2-2	5.39668116	6.29E-119	1.97E-118
TRBV7-4_TRBJ2-3	3.7388578	9.62E-96	2.67E-95
TRBV7-4_TRBJ2-4	0.52310271	4.91E-22	7.43E-22
TRBV7-4_TRBJ2-5	3.00475257	8.39E-93	2.28E-92
TRBV7-4_TRBJ2-6	3.10145683	3.00E-154	1.42E-153
TRBV7-4_TRBJ2-7	0.77537127	5.45E-29	8.76E-29
TRBV7-6_TRBJ1-1	0.83582809	1.52E-66	3.40E-66
TRBV7-6_TRBJ1-2	2.4180597	0.77358056	0.78109105
TRBV7-6_TRBJ1-3	0.07276569	1.63E-186	1.59E-185
TRBV7-6_TRBJ1-4	0.87732641	0.8492034	0.85468213
TRBV7-6_TRBJ1-5	0.84911293	1.82E-16	2.58E-16
TRBV7-6_TRBJ1-6	1.35025449	0.22445761	0.23460896
TRBV7-6_TRBJ2-1	0.53855356	5.01E-103	1.45E-102
TRBV7-6_TRBJ2-2	0.7456075	1.38E-30	2.29E-30
TRBV7-6_TRBJ2-3	0.55256941	2.16E-81	5.36E-81
TRBV7-6_TRBJ2-4	0.38226864	9.62E-13	1.28E-12
TRBV7-6_TRBJ2-5	0.6368932	6.39E-41	1.15E-40
TRBV7-6_TRBJ2-6	0.51748673	1.10E-28	1.76E-28
TRBV7-6_TRBJ2-7	0.59748115	5.49E-100	1.56E-99
TRBV7-7_TRBJ1-1	0.91579206	3.61E-18	5.21E-18
TRBV7-7_TRBJ1-2	1.77062591	5.41E-37	9.44E-37
TRBV7-7_TRBJ1-3	0.08702789	6.01E-56	1.24E-55
TRBV7-7_TRBJ1-4	0.60228448	1.03E-05	1.22E-05
TRBV7-7_TRBJ1-5	0.49235523	0.00029321	0.00033881
TRBV7-7_TRBJ1-6	8.41942326	1.95E-116	6.01E-116
TRBV7-7_TRBJ2-1	0.90668498	1.12E-64	2.45E-64
TRBV7-7_TRBJ2-2	0.69406162	2.92E-21	4.39E-21
TRBV7-7_TRBJ2-3	0.73156201	1.37E-24	2.11E-24
TRBV7-7_TRBJ2-4	0.29252828	4.13E-18	5.95E-18
TRBV7-7_TRBJ2-5	0.64873863	5.64E-45	1.07E-44
TRBV7-7_TRBJ2-6	0.3975008	1.72E-10	2.22E-10
TRBV7-7_TRBJ2-7	0.22537678	5.11E-63	1.10E-62
TRBV7-8_TRBJ1-1	1.33292225	1.92E-13	2.58E-13

TRBV7-8_TRBJ1-2	2.85040233	2.67E-128	9.00E-128
TRBV7-8_TRBJ1-3	0.17147485	6.00E-154	2.81E-153
TRBV7-8_TRBJ1-4	2.61651989	8.51E-82	2.12E-81
TRBV7-8_TRBJ1-5	1.91676606	2.88E-48	5.54E-48
TRBV7-8_TRBJ1-6	4.90868885	5.05E-175	3.35E-174
TRBV7-8_TRBJ2-1	1.33516853	0.39060884	0.4035429
TRBV7-8_TRBJ2-2	2.34254868	1.27E-77	3.07E-77
TRBV7-8_TRBJ2-3	1.48497186	0.00302459	0.00341911
TRBV7-8_TRBJ2-4	0.85340969	0.44982336	0.46242137
TRBV7-8_TRBJ2-5	1.31704537	2.29E-05	2.72E-05
TRBV7-8_TRBJ2-6	1.76817066	4.19E-10	5.38E-10
TRBV7-8_TRBJ2-7	1.43617963	0.01098622	0.01215497
TRBV7-9_TRBJ1-1	0.50577908	8.67E-125	2.86E-124
TRBV7-9_TRBJ1-2	0.91343432	6.35E-16	8.91E-16
TRBV7-9_TRBJ1-3	0.04520748	1.23E-199	4.50E-198
TRBV7-9_TRBJ1-4	0.91033938	1.23E-13	1.66E-13
TRBV7-9_TRBJ1-5	0.53741588	1.37E-76	3.25E-76
TRBV7-9_TRBJ1-6	3.26260179	9.37E-83	2.35E-82
TRBV7-9_TRBJ2-1	0.45603093	2.27E-138	8.64E-138
TRBV7-9_TRBJ2-2	0.79627654	6.47E-37	1.12E-36
TRBV7-9_TRBJ2-3	0.63350735	2.19E-99	6.22E-99
TRBV7-9_TRBJ2-4	0.31142587	3.24E-66	7.23E-66
TRBV7-9_TRBJ2-5	0.43584633	1.97E-137	7.35E-137
TRBV7-9_TRBJ2-6	0.59972392	2.09E-77	5.02E-77
TRBV7-9_TRBJ2-7	0.43705148	1.74E-145	7.02E-145
TRBV9_TRBJ1-1	0.73969786	9.76E-94	2.68E-93
TRBV9_TRBJ1-2	1.08854637	7.80E-08	9.69E-08
TRBV9_TRBJ1-3	0.04972155	2.22E-200	9.91E-199
TRBV9_TRBJ1-4	1.18323564	0.24554695	0.25579516
TRBV9_TRBJ1-5	0.72673802	1.05E-39	1.87E-39
TRBV9_TRBJ1-6	3.55775742	2.34E-62	5.04E-62
TRBV9_TRBJ2-1	0.57386401	4.11E-108	1.22E-107
TRBV9_TRBJ2-2	0.87556953	2.85E-15	3.94E-15
TRBV9_TRBJ2-3	0.56001654	1.11E-95	3.07E-95
TRBV9_TRBJ2-4	0.35486491	5.82E-44	1.08E-43
TRBV9_TRBJ2-5	0.38539037	4.93E-152	2.23E-151
TRBV9_TRBJ2-6	0.57131656	8.33E-92	2.23E-91
TRBV9_TRBJ2-7	0.69218569	9.56E-130	3.24E-129
